# Benchmarking privacy and utility in synthetic tabular cohorts for Alzheimer's disease research

**DOI:** 10.1002/dad2.70430

**Published:** 2026-08-10

**Authors:** Filip Winzell, Ida Arvidsson, Niels Christian Overgaard, Anders Heyden, Kalle Åström, Linda Karlsson, Jacob W. Vogel, Oskar Hansson, Niklas Mattsson‐Carlgren, for the Alzheimer's Disease Neuroimaging Initiative

**Affiliations:** ^1^ Centre for Mathematical Sciences Lund University Lund Sweden; ^2^ Clinical Memory Research Unit Department of Clinical Sciences in Malmö Lund University Lund Sweden; ^3^ Department of Clinical Sciences Malmö, SciLifeLab Lund University Lund Sweden; ^4^ Wallenberg Center for Molecular Medicine Lund University Lund Sweden; ^5^ Memory Clinic Skåne University Hospital Malmö Sweden

**Keywords:** Alzheimer's disease, machine learning, synthetic data, tabular data

## Abstract

**INTRODUCTION:**

The scarcity of large, clinically relevant cohorts is becoming a bottleneck in Alzheimer's disease (AD) research, as their sensitive nature makes open data sharing difficult. Privacy‐preserving synthetic datasets generated with machine learning may help address this challenge.

**METHODS:**

We compared five frameworks for generating synthetic tabular data from the Alzheimer's Disease Neuroimaging Initiative and Anti‐Amyloid Treatment in Asymptomatic Alzheimer's Disease cohorts, with a set of empirical privacy and utility metrics. Two of the methods, DataSynthesizer and TableDiffusion, provide ε‐differential privacy guarantees.

**RESULTS:**

Methods with differential privacy achieved high privacy ratings but low levels of utility. Deep learning methods like Tabular Prior‐data Fitted Network (TabPFN) and Conditional Generative Adversarial Network (CTGAN) also showed high privacy with limited utility. In contrast, non‐private DataSynthesizer and Synthpop offered higher utility at a cost of lower privacy.

**DISCUSSION:**

The evaluated methods demonstrated a clear trade‐off between privacy and utility. High privacy was generally associated with insufficient utility, highlighting the need for further research into synthetic data generation for AD.

## Highlights

- Using five popular methods, we generated synthetic tabular data for Alzheimer’s disease research.
- We benchmarked the utility and privacy with a set of well‐established and novel methods.
- Current methods do not provide satisfactory results in the utility–privacy trade‐off.

## 1 INTRODUCTION

Alzheimer's disease (AD) is the most common cause of dementia and a major contributor to cognitive decline and mortality worldwide. There is a great need for more research in this field to increase our understanding of disease mechanisms for the development of new therapies. The ability to access clinically relevant human data is a bottleneck for such research. There is especially a need for scientists to be able to access data from large and deeply phenotyped cohorts with research‐grade quality. Unfortunately, such data are difficult, time consuming, and expensive to collect, because they require advanced and expensive tools, competent and experienced staff, and vulnerable research participants motivated and able to participate. Several large relevant AD‐related datasets exist^1^, ^2^, ^3^, ^4^, ^5^, ^6^ but due to the sensitive nature of the data, they can typically only be shared among researchers after comprehensive evaluation processes and transfer agreements. Although open‐access cohorts such as the Alzheimer's Disease Neuroimaging Initiative (ADNI)^7^ and Anti‐Amyloid Treatment in Asymptomatic Alzheimer's Disease (A4)^8^ have enabled important AD research, they remain limited in size and representativeness. Therefore, a need for larger, open multi‐modal databases for AD studies remains.

Synthetic datasets offer a potential means of addressing these challenges.^9^, ^10^, ^11^, ^12^ In addition, they may also be used to augment small or imbalanced cohorts, support reproducible benchmarking, and simulate disease trajectories.^12^, ^13^ Such datasets need to fulfill key requirements, such as preserving both the privacy of the real patient data used to develop them and the natural relationships among phenotypes, to the point that models trained on these synthetic datasets can generalize to real, external datasets. Numerous statistical and deep learning–based methods exist for generating synthetic tabular data.^13^, ^14^, ^15^, ^16^, ^17^, ^18^ Still, there is a lack of studies that systematically evaluate and compare methods to generate synthetic AD cohorts.

In this work, we benchmarked several state‐of‐the‐art methods for generating synthetic tabular AD data. We evaluated their capabilities of privacy protection, preservation of phenotype relationships, and downstream utility for machine learning across several simple tasks. Our code used in this project is openly accessible at https://github.com/fwinzell/synthetic_ad.

## 2 METHODS

**FIGURE 1:**
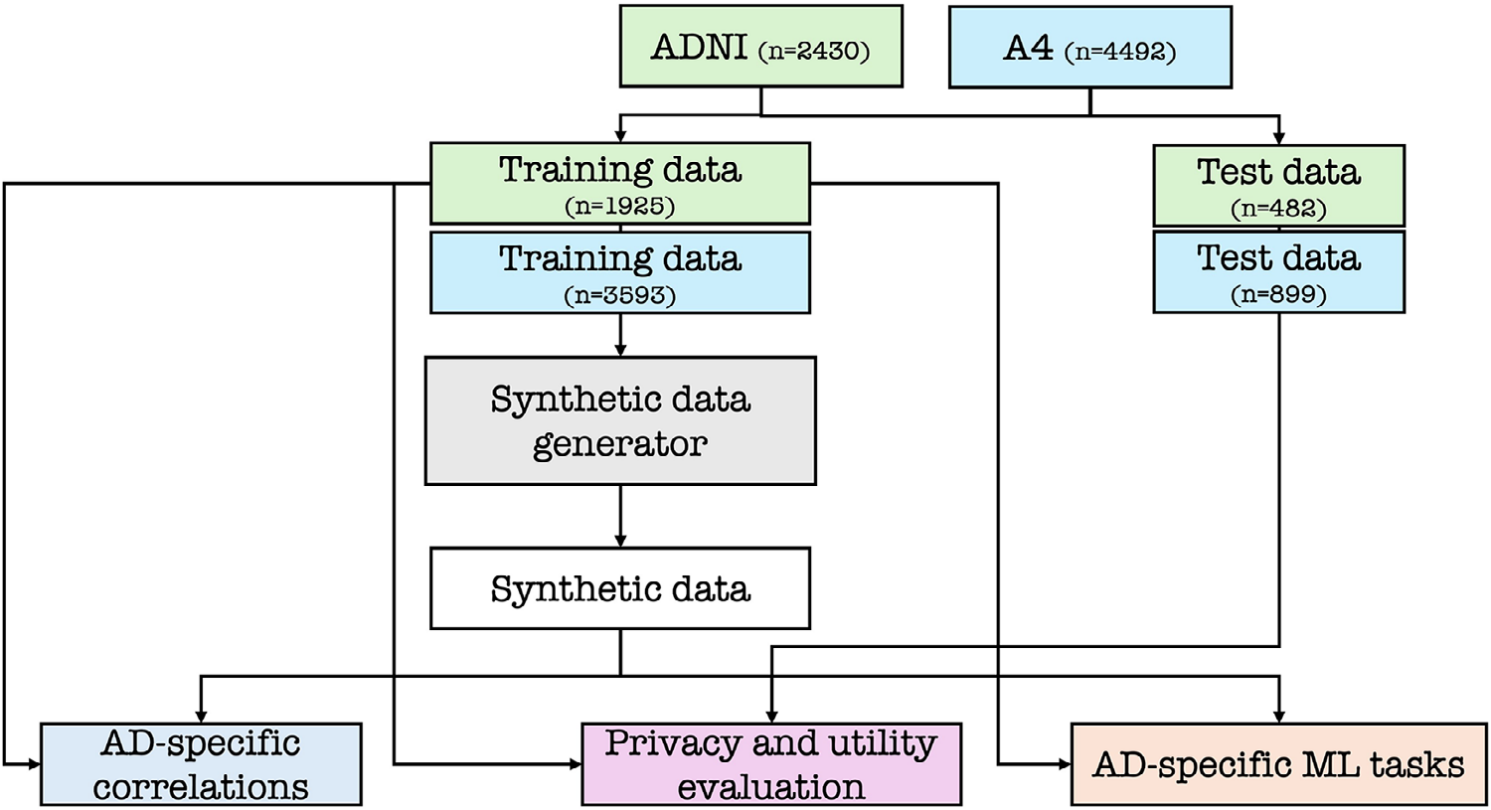
Overview of our methodology. We sampled a subset of 18 attributes from the baseline data of 2430 participants of ADNI, and 23 attributes from 4492 participants of A4. We split this dataset into a training and hold‐out test set. The training data were used to generate synthetic data and as a benchmark for evaluation. We evaluated the generated synthetic data by studying AD‐specific known correlations, using privacy and utility metrics, and by training and testing AD‐specific machine learning algorithms. A4, Anti‐Amyloid Treatment in Asymptomatic Alzheimer's Disease; AD, Alzheimer's disease; ADNI, Alzheimer's Disease Neuroimaging Initiative; ML, machine learning.

We leveraged data from ADNI and A4, and five frameworks (DataSynthesizer [DS], Conditional Generative Adversarial Network [CTGAN], Tabular Prior‐data Fitted Network [TabPFN], Synthpop, and TableDiffusion) to develop synthetic cohorts. We designed a pipeline to fine‐tune and evaluate the synthetic cohort in a manner that was specific and relevant to AD studies (see Figure 1).

**RESEARCH IN CONTEXT**

1. **Systematic review**: The authors used common methods such as Google Scholar searches to find relevant sources for the literature review. Generation of synthetic tabular data is a field that has received considerable research interest; however, it has not yet been applied to the field of Alzheimer's disease (AD) research.
2. **Interpretation**: We present a first step toward generating a full synthetic cohort of research‐grade AD data. We discuss the major limitations of different popular methods.
3. **Future directions**: Our framework for evaluating and benchmarking synthetic AD datasets can be used by future studies and serves as a baseline. Future research involves developing novel methods for synthetic data generation that can both achieve satisfactory utility and privacy ratings and incorporate more variables with longitudinal measurements.

### 2.1 Data

Data used in the preparation of this article were obtained from the ADNI database and the A4 study. From ADNI, we used the baseline data from a subset of 18 clinically relevant attributes, including demographic variables, cerebrospinal fluid (CSF) and positron emission tomography (PET) imaging biomarkers, magnetic resonance imaging (MRI) measurements, cognitive tests, and baseline diagnosis.

From the A4 study, we combined baseline measurements for 23 of the demographic variables, assessments, and imaging biomarkers. Both cohorts were randomly split at patient level by 80%/20% into a training and hold‐out test set. See Table SA.1 in supporting information for a summary of the cohorts.

We also investigated the inclusion of additional imaging biomarkers in the ADNI cohort. In this cohort, which we denote ADNI+, we replaced the original 7 MRI biomarkers with 35 volumetric MRI variables from the University of California San Francisco cross‐sectional dataset (see Table SA.2 in supporting information).

We applied the pre‐processing steps outlined in supporting information (Appendix SA.2) to all three cohorts.

### 2.2 Generating synthetic tabular data

DS is a tool for generating privacy‐preserving synthetic datasets based on Bayesian networks.^14^ It uses the GreedyBayes algorithm (summarized in Appendix SA.3 in supporting information)^19^ to construct the Bayesian network, with the possibility of using ε‐differential privacy (ε‐DP) constraints. An algorithm is said to fulfill ε‐DP constraints if changing a tuple of the input dataset does not change the probability of getting the same output by more than a ratio of $e^{\varepsilon }$. In other words, the output probability distribution is independent (to a degree specified by ε) from specific tuples of the input data. We refer to the original publications for additional details on this.^14^, ^19^ TableDiffusion is a more recent method for generating synthetic tabular data with ε‐DP.^18^ It is based on a diffusion model and uses the differential privacy stochastic gradient descent (DP‐SGD) optimizer to ensure ε‐DP.

We used DS in correlated attribute mode with default settings, where we constructed 100 Bayesian networks for the ε‐levels ε∈[200,100,50,10,5], and subsequently generated one dataset for each with the same number of tuples as the real train split ($n_{ADNI}=1925$, $n_{A4}=3593$). Note that a lower ε means increased privacy. We also constructed networks (one for each possible random root) without ε‐DP activated. In an initial experiment, we found that using a degree of 2 for the networks would be optimal (see Figure SB.1 in supporting information). For TableDiffusion, we used the ε‐levels ε∈[100,50,25,10,1], and as suggested by Truda, we used the Optuna library for hyperparameter tuning for each level and dataset separately.^18^

We also generated synthetic datasets with larger deep learning‐based models, namely the GAN‐based method CTGAN,^15^ and the tabular foundation model TabPFN.^17^ We evaluated hyperparameters for CTGAN with a cross‐validation loop, in which we used the synthetic datasets to train a simple machine learning (ML) model on predicting baseline diagnosis (same approach as described in section 2.4.1). We compared CTGAN with the optimized setting to the default hyperparameters. To investigate whether these parameters would translate across cohorts, we used the same values when training on A4. TabPFN is a generative transformer‐based model, aimed at generalizing to any tabular dataset. It was developed for classification and regression tasks, but can also be used to generate full synthetic datasets. We evaluated four different settings for the temperature sampling parameter (t=[0.25,0.5,0.75,1.0]), in which a higher temperature means less deterministic samples. Last, we adopted the R implementation of CARTs for synthesizing tabular data, Synthpop.^16^

In a final experiment, we evaluated the effect of including additional imaging biomarkers by evaluating a subset of the methods on the ADNI+ cohort. Specifically, DS (ε=100), DS without ε‐DP, Synthpop, CTGAN (default), and TabPFN (*t* = 1.0).

### 2.3 Privacy evaluation

Quantifying the privacy of the synthetic datasets is naturally an integral part of this study. To facilitate benchmarking of synthetic tabular datasets, we used several metrics from the open‐source framework SynthEval.^20^ Specifically, we used nearest neighbor adversarial accuracy (NNAA) and nearest neighbor distance ratio (NNDR). As recommended, we report privacy loss^20^ for these metrics, defined as the difference between the metric values computed on the training (or synthetic) dataset and the hold‐out test dataset.^19^ In addition to these metrics, we also used hitting rate, ε‐identifiability risk, membership inference attack (MIA), and attribute disclosure risk (ADR) from the SynthEval package (see Table SA.3 in supporting information). All privacy metrics are detailed in supporting information (Appendix SA.4).

### 2.4 Utility evaluation

Achieving high privacy scores is irrelevant if the utility of the data is insufficient. To benchmark utility, we used the difference in mutual information between synthetic and real datasets, the Kolmogorov–Smirnov (KS)/total variation distance (TVD) test, and the difference in F1 score metrics from SynthEval. The F1 metric evaluates four different ML‐based classifiers’ capabilities to classify real unseen samples on the validation folds and on the hold‐out test set. As suggested by Lautrup et al.,^21^ we summarized the metrics into average privacy and utility scores for each synthetic dataset (see Eq. SA.3–4 in supporting information). More details on the SynthEval metrics are provided in Appendix SA.4, and they are summarized in Table SA.3.

Given our specific application of synthetic tabular data generation in AD research, in addition to the metrics in SynthEval, we implemented some AD‐specific metrics, based on known correlations and previous research. We compared Pearson correlation coefficient between the cognitive scores (Alzheimer's Disease Assessment Scale Cognitive part 13‐item version [ADAS13] and Mini‐Mental State Examination [MMSE]) and the biomarkers total tau (TAU) and phosphorylated tau (PTAU) in the ADNI cohort. For A4 we used the following combinations of three cognitive tests; MMSE, Logical memory (immediate and delayed recall), and Digit Symbol Substitution Test. We also compared the percentages of amyloid beta (Aβ) positives (for ADNI; using the cut‐off point of > 1.11 for ^18^F, and for A4, the generated Aβ status) and the ratio of apolipoprotein E (*APOE*) ε4 carriers within the diagnostic groups.

#### 2.4.1 ML

Furthermore, we trained ML models to predict baseline diagnosis (ADNI), Aβ positivity, and cognitive status (MMSE and ADAS13 scores). Specifically, we trained support vector machine (SVM) and histogram gradient‐boosting tree (HGBoost) classifiers for the former two and regression models for the latter. In contrast to neural networks, these methods are easier to optimize on smaller datasets and do not depend as heavily on hyperparameters and architectural choices. We included all variables except diagnosis and the predictor at hand (excluding both cognitive tests for both tasks). A 6‐fold cross‐validation grid search was used to find the optimal hyperparameters for each ML model in each setting, in both cohorts. We compared training these algorithms on real and synthetic data and evaluated them on our hold‐out test set of real data.

#### 2.4.2 Missing values

In any multi‐modal dataset, missing values will likely occur, and therefore, realistic synthetic data will also contain missing values. Because only the HGBoost algorithm can handle missing values, we imputed the missing values using scikit‐learn's IterativeImputer and SimpleImputer for numerical and categorical attributes, respectively.^22^ This approach was also applied to the training data for CTGAN and TabPFN, as these methods do not natively handle missing values.

## 3 RESULTS

The cohorts and their training and test splits, after running the pre‐processing steps in Appendix SA.3, are summarized in Table SA.1. The results of the hyperparameter search for CTGAN and TableDiffusion can be found in Tables SB.4–SB.5 in supporting information.

### 3.1 Privacy analysis

**FIGURE 2:**
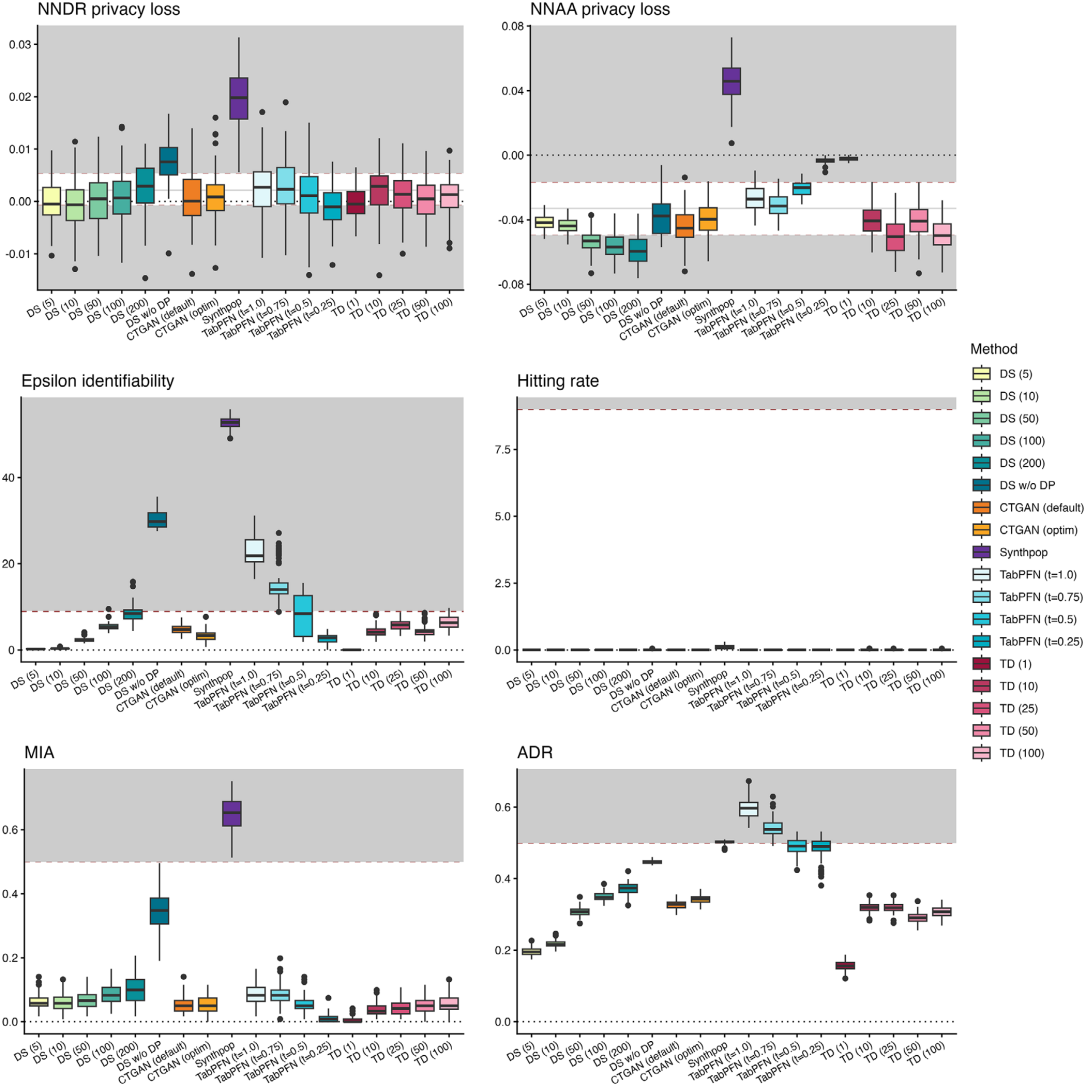
Privacy evaluation with SynthEval for the synthetic datasets generated based on the ADNI cohort. On the *x* axis: DS (with ε in parentheses) and without DP, CTGAN (with number of epochs in parentheses), Synthpop, TabPFN (with temperature in parentheses), and TD (with ε in parentheses). For all metrics, a lower value corresponds with better privacy. The boxplots visualizes median (middle line), the IQR from the 25th to the 75th percentile (box), 1.5xIQR (whiskers), and any outliers. For the DS datasets, summary statistics were estimated by the 100 constructed Bayesian networks for each epsilon setting (18/23 for non ε‐DP). For the CTGAN and Synthpop datasets, 100 different datasets generated with different seeds were used for statistical evaluation. For the top row, the gray line and dashed red lines represent the global mean and 99% CI, estimated using a linear mixed‐effects model with a random intercept for method to account for between‐method variability. For the middle and bottom rows, gray areas indicate the acceptable thresholds of 9% and 0.5, respectively. ADNI, Alzheimer's Disease Neuroimaging Initiative; ADR, attribute disclosure risk; CI, confidence interval; CTGAN, Conditional Generative Adversarial Network; DP, differential privacy; DS, DataSynthesizer; IQR, interquartile range; MIA, membership inference attack; NNAA, nearest neighbor adversarial accuracy; NNDR, nearest neighbor difference ratio; TabPFN, Tabular Prior‐data Fitted Network; TD, TableDiffusion.

**FIGURE 3:**
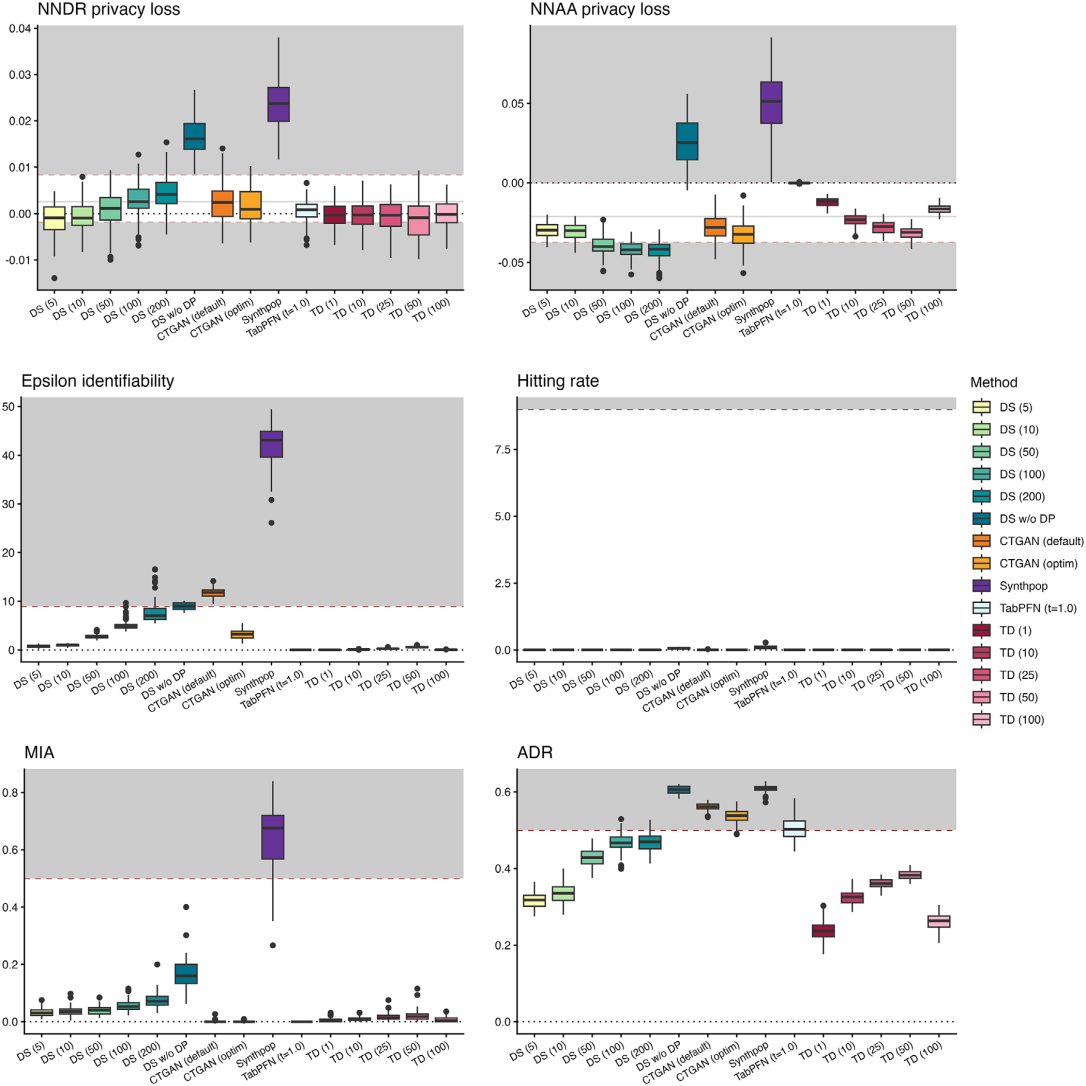
Privacy evaluation with SynthEval for the synthetic datasets generated based on the A4 cohort. On the *x* axis: DS (with ε in parentheses) and without DP, CTGAN (with number of epochs in parentheses), Synthpop, TabPFN (with temperature in parentheses), and TD (with ε in parentheses). For all metrics, a lower value corresponds with better privacy. The boxplot visualizes median (middle line), the IQR from the 25th to the 75th percentile (box), 1.5xIQR (whiskers), and any outliers. For the DS datasets, summary statistics were estimated by the 100 constructed Bayesian networks for each epsilon setting (18/23 for non ε‐DP). For the CTGAN and Synthpop datasets, 100 different datasets generated with different seeds were used for statistical evaluation. For the top row, the gray line and dashed red lines represent the global mean and 99% CI, estimated using a linear mixed‐effects model with a random intercept for method to account for between‐method variability. For the middle and bottom rows, gray areas indicate the acceptable thresholds of 9% and 0.5, respectively. A4, Anti‐Amyloid Treatment in Asymptomatic Alzheimer's Disease; ADR, attribute disclosure risk; CI, confidence interval; CTGAN, Conditional Generative Adversarial Network; DP, differential privacy; DS, DataSynthesizer; IQR, interquartile range; MIA, membership inference attack; NNAA, nearest neighbor adversarial accuracy; NNDR, nearest neighbor difference ratio; TabPFN, Tabular Prior‐data Fitted Network; TD, TableDiffusion.

The results of the empirical privacy metrics are presented in Figures 2, 3. For ε‐identifiability risk and hitting rate, it is mentioned in the literature that a score < 9% is a good aim.^20^ The Synthpop datasets failed this by a large amount for the ε‐identifiability test with mean values of 52.6% (95% confidence interval [CI]: 52.3–52.9) and 42.3% (95% CI: 41.4–43.1), for ADNI and A4, respectively. In addition, the non‐DP DS also failed this on ADNI (95% CI: 29.0–31.3) and partially on A4 (95% CI: 8.7–9.3). For MIA and ADR, a threshold of random guessing (0.5) is often used.^23^ Consistent with previous results, the Synthpop datasets scored average recall values above this on both ADNI (95% CI: 0.638–0.660) and A4 (95% CI: 0.625–0.672) for the MIA test. The non‐DP DS datasets were below the MIA threshold, but with significantly higher values than the other methods (non‐overlapping 95% CIs) for both cohorts. On the ADR, Synthpop, TabPFN (*t* = 1.0, *t *= 0.75), and CTGAN on A4 were above the threshold. The non‐DP DS passed on ADNI but not on A4 (95% CI: 0.600–0.609).

### 3.2 Utility analysis

The results on the utility metrics are presented in Figures SB.2–SB.3 in supporting information. Here, we can observe the opposite relationship, where the ε‐DP, CTGAN, and TabPFN datasets scored considerably worse than the other non‐DP DS and Synthpop datasets.

**FIGURE 4:**
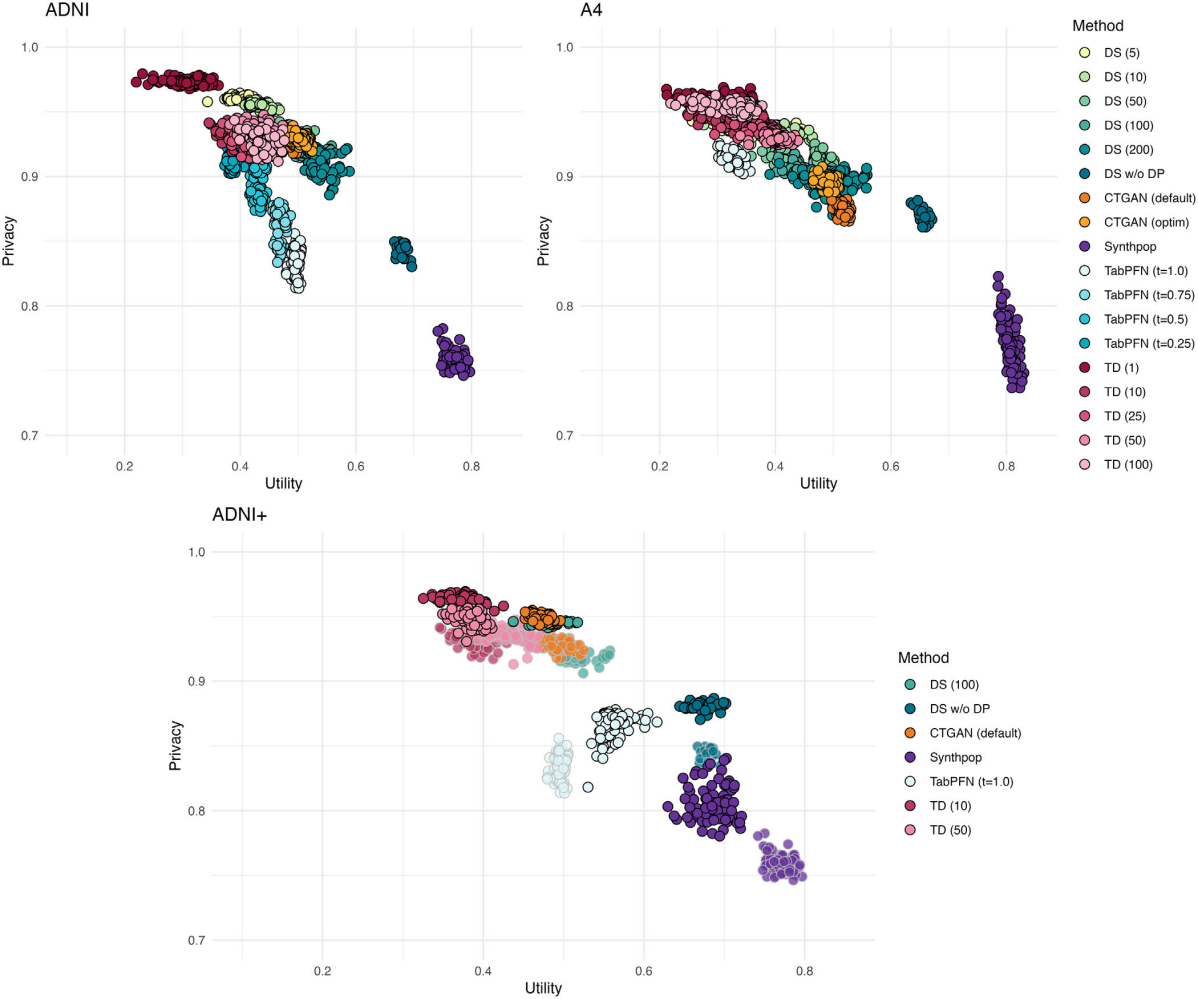
Privacy versus utility score. Evaluated for the synthetic datasets generated using DS with (ε in parenthesis) and without DP, CTGAN (with number of epochs in parenthesis), Synthpop, TabPFN (temperature in parentheses), and TD (ε in parenthesis). The top row shows the results on the ADNI and A4 cohorts. The bottom shows the ADNI+ results, with the corresponding results from ADNI shaded in the background. A4, Anti‐Amyloid Treatment in Asymptomatic Alzheimer's Disease; ADNI, Alzheimer's Disease Neuroimaging Initiative; CTGAN, Conditional Generative Adversarial Network; DP, differential privacy; DS, DataSynthesizer; IQR, interquartile range; TabPFN, Tabular Prior‐data Fitted Network; TD, TableDiffusion.

We summarized the metrics into a utility and privacy score using Equations 1 and 2 for both cohorts (see Figure 4). On the ADNI cohort, the highest average privacy scoring dataset, TableDiffusion ε=1 (0.973, 95% CI: 0.972–0.974), scored the lowest utility score with 0.310 (95% CI: 0.304–0.316). Notably, the second lowest utility on the A4 cohort was TableDiffusion with ε=100. All datasets seemed to follow a similar trajectory of privacy–utility trade‐off, for which Synthpop achieved the highest utility (ADNI: 0.770, A4: 0.806) and lowest privacy (ADNI: 0.759, A4: 0.769). Finally, we investigated the inclusion of several additional imaging variables in the ADNI+ cohort (see Figure 4). This significantly improved the privacy scores for all tested methods (*P* < 0.001 for all, evaluated with a Welch *t*‐test with Bonferroni correction) compared to the ADNI cohort. It had mixed effects on the utility, with improved scores for the TabPFN (*t* = 1.0) datasets (*P* < 0.001), and reduced scores for DS (ε=100; *P* < 0.001), CTGAN (*P* < 0.001), TableDiffusion (*P* < 0.001), and Synthpop (*P* < 0.001).

**FIGURE 5:**
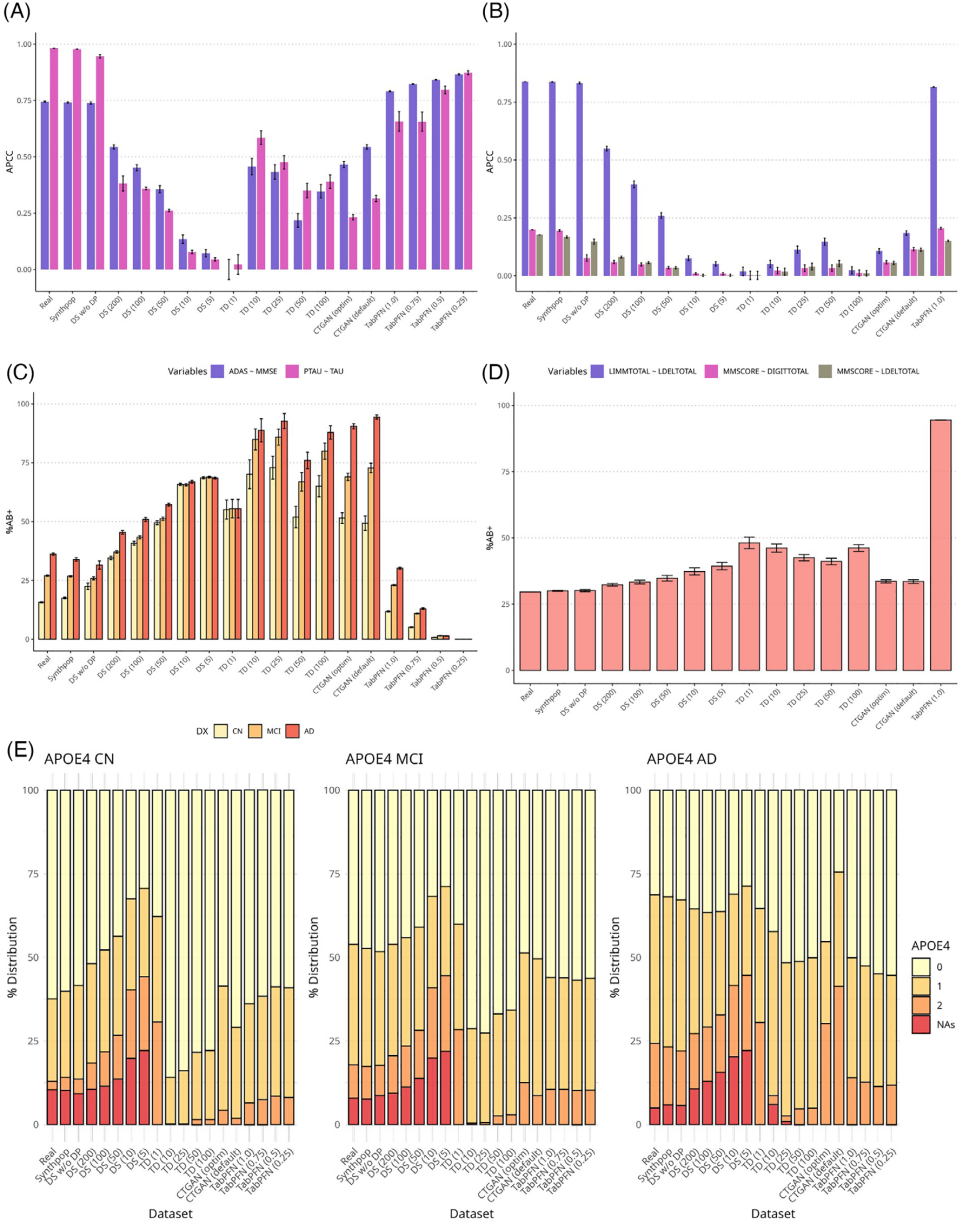
Visualization of different characteristics of the synthetic datasets compared to the real. A, Absolute Pearson correlation coefficient (APCC) between the cognitive tests ADAS13 and MMSE and between the biomarkers PTAU and TAU on ADNI. B, APCC between the cognitive tests Logical Memory Immediate (LIMM), Logical Memory Delayed (LDEL), MMSE, and Digit Symbol Substitution Test (DIGIT) on the A4 study. C, Percentage of A β positive subjects within each diagnostic group on ADNI. Positivity was defined as subjects with abnormal A β PET (FBP SUVR > 1.11). D, Percentage of A β positive subjects on A4, encoded as an independent categorical variable. E, Distribution of *APOE* ε 4 alleles for the diagnostic groups on ADNI. A β, amyloid beta; A4, Anti‐Amyloid Treatment in Asymptomatic Alzheimer's Disease; ADAS13, Alzheimer's Disease Assessment Scale Cognitive part 13‐item version; ADNI, Alzheimer's Disease Neuroimaging Initiative; *APOE*, apolipoprotein E; CTGAN, Conditional Generative Adversarial Network; DS, DataSynthesizer; FBP, florbetapir; MMSE, Mini‐Mental State Examination; PET, positron emission tomography; PTAU, phosphorylated tau; SUVR, standardized uptake value ratio; TabPFN, Tabular Prior‐data Fitted Network; TAU, total tau; TD, TableDiffusion.

For the AD‐specific correlations and distributions, we saw a similar pattern, in which the quality of the synthetic declined significantly with added ε ‐DP constraints (see Figure 5). Synthpop and the non‐DP DS datasets managed to capture these correlations close to the real data. Interestingly, on the ADNI cohort, the TabPFN datasets showed realistic correlations, and the t = 1.0 version managed to produce A β + and *APOE* ε 4 distributions close to the real data. On A4, the correlations of the cognitive test were realistic, but the A β + status was at 94.4% (95% CI: 94.4–94.5) compared to the real data, with 30.0%.

### 3.3 ML algorithms

Results of the ML algorithms on the different synthetic datasets are presented in Table 1 (see Table SB.6 in supporting information for the resulting hyperparameters). We used the 95% CI of the difference between the synthetic and the real training data to assess statistical significance. The Synthpop datasets consistently provided strong results, which were not significantly worse than the real datasets for A β+ prediction on ADNI and MMSE regression on A4 using the SVM model. Surprisingly, the TabPFN (*t* = 1.0) datasets gave the strongest performance on the A β+ task on ADNI (0.880, 95% CI: 0.879–0.882). In a post hoc analysis, fitting a linear SVM on this task produced high weights to the CSF‐based biomarkers A $\beta_{42}$ and PTAU for these datasets, indicating that TabPFN managed to capture those distributions realistically. The non‐DP DS datasets gave consistently strong performances; however, they only achieved a non‐significant difference with respect to the real data on the A β+ task of A4.

**TABLE 1:** The average scores of the two models (SVM and HGBoost) on the four tasks; diagnosis classification (DX), A β status classification (A β), ADAS13, and MMSE prediction. Classification performance was evaluated with ROC curve AUC (1‐vs.‐the‐rest AUC for DX), averaged over the constructed datasets.

Method Dataset		ADNI				A4	
	ε/n/t	DX (1 vs. r. AUC) ↑	A β+(AUC) ↑	ADAS13 (RMSE) ↓	MMSE (RMSE) ↓	A β+ (AUC) ↑	MMSE (RMSE) ↓
**SVM**							
Real		**0.869** (0.869–0.870)	**0.868** (0.868–0.869)	**7.778** (7.749–7.808)	**2.249** (2.241–2.258)	**0.947** (0.945–0.948)	**1.182** (1.180–1.184)
DS	5	0.650 (0.635–0.666)	0.513 (0.493–0.534)	12.023 (11.71–12.34)	4.537 (4.395–4.678)	0.717 (0.700–0.734)	1.339 (1.316–1.361)
DS	10	0.720 (0.708–0.731)	0.540 (0.517–0.563)	10.767 (10.49–11.04)	4.047 (3.912–4.182)	0.735 (0.724–0.747)	1.309 (1.292–1.326)
DS	50	0.843 (0.841–0.845)	0.679 (0.656–0.701)	8.651 (8.597–8.705)	2.575 (2.554–2.595)	0.787 (0.777–0.796)	1.200 (1.197–1.202)
DS	100	0.852 (0.850–0.853)	0.741 (0.724–0.758)	8.323 (8.282–8.363)	2.467 (2.453–2.481)	0.827 (0.819–0.835)	1.192 (1.190–1.194)
DS	200	0.855 (0.854–0.856)	0.776 (0.763–0.789)	8.204 (8.166–8.241)	2.444 (2.431–2.457)	0.838 (0.829–0.848)	1.189 (1.188–1.191)
DS		0.857 (0.854–0.860)	0.860 (0.855–0.865)	7.892 (7.812–7.972)	2.345 (2.322–2.368)	0.944* (0.938–0.950)	1.188 (1.185–1.192)
TableDiff	1	0.537 (0.519–0.554)	0.544 (0.515–0.573)	16.124 (14.99–17.26)	4.958 (4.601–5.316)	0.521 (0.510–0.531)	1.960 (1.868–2.052)
TableDiff	10	0.680 (0.668–0.691)	0.726 (0.700–0.752)	11.396 (11.01–11.78)	2.816 (2.674–2.958)	0.527 (0.515–0.538)	1.708 (1.645–1.772)
TableDiff	25	0.683 (0.669–0.696)	0.721 (0.699–0.744)	11.687 (11.32–12.05)	2.859 (2.741–2.977)	0.535 (0.524–0.546)	1.589 (1.535–1.643)
TableDiff	50	0.699 (0.688–0.710)	0.732 (0.719–0.745)	12.255 (11.86–12.64)	3.389 (3.202–3.577)	0.557 (0.548–0.566)	1.475 (1.436–1.514)
TableDiff	100	0.702 (0.691–0.713)	0.750 (0.734–0.766)	11.682 (11.27–12.08)	3.311 (3.114–3.507)	0.505 (0.494–0.516)	2.053 (1.973–2.134)
CTGAN	750	0.798 (0.796–0.801)	0.764 (0.749–0.779)	8.597 (8.439–8.756)	2.375 (2.364–2.386)	0.880 (0.876–0.885)	1.221 (1.212–1.229)
Synthpop		**0.863** (0.862–0.865)	0.868* (0.866–0.870)	**7.860** (7.812–7.907)	**2.275** (2.265–2.284)	0.942 (0.939–0.945)	1.181* (1.179–1.183)
TabPFN	1.0	0.762 (0.756–0.769)	0.880* (0.878–0.882)	10.359 (9.947–10.77)	3.060 (2.913–3.208)	0.539 (0.528–0.549)	24.202 (24.17–24.24)
**HGBoost**							
Real		**0.869** (0.869–0.870)	**0.877** (0.875–0.879)	**7.500** (7.482–7.518)	**2.310** (2.303–2.317)	**0.983** (0.982–0.983)	1.187 (1.184–1.189)
DS	5	0.728 (0.718–0.739)	0.523 (0.504–0.543)	12.915 (12.59–13.24)	4.702 (4.589–4.815)	0.528 (0.502–0.555)	1.503 (1.480–1.527)
DS	10	0.779 (0.772–0.786)	0.562 (0.544–0.579)	11.554 (11.28–11.83)	4.356 (4.230–4.481)	0.554 (0.525–0.583)	1.439 (1.418–1.460)
DS	50	0.833 (0.831–0.835)	0.697 (0.681–0.713)	9.208 (9.079–9.338)	3.056 (3.003–3.109)	0.589 (0.568–0.611)	1.212 (1.209–1.216)
DS	100	0.837 (0.834–0.839)	0.750 (0.737–0.762)	8.752 (8.670–8.834)	2.692 (2.663–2.722)	0.650 (0.625–0.675)	1.193 (1.191–1.195)
DS	200	0.835 (0.833–0.837)	0.765 (0.753–0.778)	8.606 (8.545–8.668)	2.582 (2.559–2.604)	0.630 (0.605–0.655)	1.186* (1.184–1.187)
DS		0.841 (0.839–0.843)	0.842 (0.832–0.851)	8.012 (7.913–8.112)	2.407 (2.384–2.431)	0.754 (0.692–0.815)	1.184* (1.180–1.188)
TableDiff	1	0.541 (0.525–0.556)	0.535 (0.509–0.561)	15.849 (14.99–16.70)	4.833 (4.537–5.129)	0.514 (0.493–0.534)	1.955 (1.879–2.031)
TableDiff	10	0.656 (0.646–0.666)	0.644 (0.620–0.667)	10.691 (10.48–10.90)	2.765 (2.662–2.869)	0.538 (0.521–0.555)	1.782 (1.732–1.831)
TableDiff	25	0.660 (0.650–0.670)	0.639 (0.618–0.660)	10.608 (10.39–10.82)	2.762 (2.685–2.839)	0.534 (0.517–0.552)	1.663 (1.618–1.708)
TableDiff	50	0.673 (0.663–0.683)	0.672 (0.656–0.688)	11.299 (11.03–11.56)	3.428 (3.254–3.602)	0.550 (0.530–0.570)	1.579 (1.545–1.613)
TableDIff	100	0.675 (0.665–0.685)	0.692 (0.675–0.709)	10.712 (10.45–10.96)	3.274 (3.088–3.461)	0.515 (0.498–0.531)	1.983 (1.924–2.042)
CTGAN	750	0.803 (0.799–0.806)	0.770 (0.762–0.779)	8.842 (8.707–8.977)	2.412 (2.399–2.425)	0.765 (0.742–0.787)	1.235 (1.222–1.248)
Synthpop		**0.855** (0.853–0.857)	**0.850** (0.848–0.853)	**7.922** (7.885–7.959)	**2.368** (2.359–2.376)	**0.863** (0.842–0.884)	1.180* (1.178–1.182)
TabPFN	1.0	0.752 (0.747–0.758)	0.622 (0.601–0.644)	11.023 (10.68–11.36)	2.918 (2.846–2.990)	0.551 (0.535–0.567)	24.156 (24.12–24.19)

*Notes*: Performance on the regression tasks was evaluated with average root mean squared error (RMSE). Bold font indicates the two best‐performing scores (e.g., the real and one synthetic), and all scores are presented with 95% CIs in parentheses.* indicates that the difference with reference to the Real result was not significantly different from zero at a 95% confidence level.

Abbreviations: Aβ, amyloid beta; A4, Anti‐Amyloid Treatment in Asymptomatic Alzheimer's Disease; ADAS13, Alzheimer's Disease Assessment Scale Cognitive part 13‐item version; ADNI, Alzheimer's Disease Neuroimaging Initiative; AUC, area under the curve; CTGAN, Conditional Generative Adversarial Network; DS, DataSynthesizer; HGBoost, histogram gradient‐boosting tree; MMSE, Mini‐Mental State Examination; ROC, receiver operating characteristic; SVM, support vector machine; TabPFN, Tabular Prior‐data Fitted Network; TD, TableDiffusion.

## 4 DISCUSSION

The methods with formal ε‐DP guarantees, DS with ε‐DP and TableDiffusion, achieved high empirical privacy ratings but showed low utility. CTGAN, TabPFN, Synthpop, and non‐private DS do not provide formal ε‐DP guarantees and were therefore evaluated using the empirical privacy metrics only. Among these, Synthpop and non‐private DS offered higher utility, but with lower empirical privacy protection. Adding this constraint to the DS Bayesian networks introduces noise into the modeled data distributions. This sampling does not account for key relationships within the data, which likely explains the reduced utility. TableDiffusion adopts the DP‐SGD optimization strategy to ensure ε‐DP, which does not introduce any new noise to the data. However, all the larger, neural network–based models scored high privacy and low utility ratings, indicating that these models cannot be trained sufficiently on this limited sample size. The hyperparameters of CTGAN did not translate across the cohorts, for which the default parameters gave a better performance on the A4 cohort.

The distributions of AD‐specific biomarkers highlight the information loss in the synthetic cohorts with low utility (see Figure 5). The addition of stricter ε‐DP constraints made distributions increasingly uniform. This effect created another failure mode, in which some datasets saw A β positivity climb toward 100% on ADNI, due to the A β PET measurements converging toward a mean above the positivity cut‐off (see Figure 5C). Overall, this explains the results on the AD‐specific ML tasks, in which only 7 out of 168 synthetic dataset‐task combinations were not significantly worse than the real data (see Table 1).

While Synthpop and non‐private DS datasets preserved the correlations, this came at a cost of privacy, which can be considered unacceptable for highly sensitive variables. For instance, approximately half of the real data records have a Synthpop generated synthetic participant closer than another real, indicating that this method does not learn the key relationships but merely generates close copies of the real records. Evaluating privacy and defining acceptable privacy levels for openly sharing synthetic datasets remains challenging. While the MIA can indicate potential sensitive information leakage, it does not represent a realistic scenario because the pool of non‐training subjects would be much larger in practice. Similarly, the ADR and ϵ‐identifiability risk represent situations in which an adversary already has access to a large portion of the training data. The hitting rate can give a good lower bound of privacy, but it depends on the threshold hyperparameter for numerical attributes. In summary, these are good measures for comparing the privacy levels of synthetic datasets, yet it is difficult to draw any conclusions on what synthetic data could be shared openly. While the ϵ‐differential privacy constraint is a way to guarantee a certain privacy level mathematically, we cannot assert that this constraint is required for openly shared synthetic data.

Generating larger synthetic datasets may improve downstream ML performance only if the generator accurately captures the real data distribution. Otherwise, larger synthetic samples may amplify artifacts and complicate benchmarking, which is why we kept real and synthetic dataset sizes equal, as recommended by Lautrup et al.^20^

A further consideration is the potential for bias propagation in synthetic datasets. The synthetic data will carry biases present in the training data, which can cause discrimination based on demographics or disease severity.^24^ Furthermore, adjusting to these biases creates a bias–utility trade‐off, in which some biases might be useful for downstream performance. Future work should assess the biases in the synthetic cohorts, such as disease stage, age, sex, and biomarker status.

AD is a complex disease, and generating research‐grade synthetic datasets is not an easy task. We only selected a small subset of all the biomarkers available in current research and only focused on baseline data, ignoring the fundamental aspect of longitudinal changes. Still, for this simplified setting, we experienced difficulties in generating privacy‐protected synthetic datasets with sufficient utility. This can be explained by the limited size of our dataset, both in the number of records and attributes. To achieve high utility, some synthetic records will inevitably lie close to the real, increasing privacy risks. Enforcing hard constraints on the synthetic‐to‐real distances would make it possible to identify real records as the missing combinations on large datasets. Our experiment on the ADNI+ cohort suggests that adding attributes could be a solution, which makes it more difficult to identify specific records. However, we also saw a utility drop for all methods except non‐DP DS and TabPFN.

Federated learning is another way of mitigating issues related to data sharing when developing ML algorithms, in which you share the algorithms rather than the data between research institutions. Still, there are some privacy concerns here, which involve similar issues as with synthetic data of identifying characteristics of subjects used in the training process.^25^ Despite this, there have been several efforts at using federated learning for AD research.^26^, ^27^, ^28^ Mitrovska et al.^26^ developed a federated learning approach with secure aggregation for the detection of AD with 3D CNNs using 3D MRI data. Lei et al. achieved state‐of‐the‐art performance on AD detection from structural MRI images with a federated learning approach.^28^ While these studies show strong results, they are still only proofs of concept, in the sense that they have access to all data locally. To our knowledge, no larger studies have used federated learning to mitigate data sharing issues in AD research. Organizing a federated learning solution in practice involves tedious planning between institutions, which needs to be done for every subsequent study. Generating a synthetic dataset that can be shared openly avoids this laborious work and enables researchers to conduct several studies independently of each other.

### 4.1 Limitations

There are some limitations of this study worth mentioning. First, we have only adopted a small subset of the numerous methods for synthetic tabular data generation available.^13^, ^18^ We also only evaluated our methods on smaller subsets of the large multimodal cohorts, ADNI and A4. To leverage more tabular data, we included the foundation model TabPFN. However, it is worth noting that the model was originally developed for classification and regression tasks on large tabular datasets, not synthetic data generation.

We evaluated the synthetic datasets with classical ML models and did not introduce any deep learning–based models. To fully evaluate the downstream utility of a synthetic cohort, several neural baselines would have to be included. However, we believe that the models used in this study are sufficient to support the conclusions.

Last, the CTGAN, TabPFN, and TableDiffusion methods have no native way of handling missing values and thus need imputation. Hence, the performance of these models will also depend on the choice of the imputation function.

## 5 CONCLUSION

In response to the growing demand for large, openly shared datasets for AD research, we explored several techniques for generating synthetic tabular cohorts. This poses a trade‐off between preserving the privacy of the real patient data and maintaining the utility that is required for meaningful research. Importantly, DS and TableDiffusion enable DP guarantees and should be distinguished from the others, which can only be evaluated with empirical privacy metrics. Our results showed that only DS without ε‐DP constraints and Synthpop generated datasets with sufficient utility, however, at a cost of lower privacy ratings. TabPFN produced datasets that captured a few realistic distributions but overall scored poorly on utility. We can conclude that more work is needed to both improve the utility of the synthetic datasets while keeping the privacy of the data at an acceptable level. Furthermore, to generate a clinically relevant synthetic cohort, the methods need to be extended to model both longitudinal relationships, within different trajectories of AD, together with higher dimensionality and more complex attributes.

## CONFLICT OF INTEREST STATEMENT

The authors declare that there are no competing interests that could have influenced the objectivity of this research in this paper. N.M.C. has received speaker/consulting fees from Biogen, BioArctic, Eli Lilly, Novo Nordisk, Owkin, and Merck. O.H. is an employee of Lund University and Eli Lilly. Author disclosures are available in the Supporting Information.

## CONSENT STATEMENT

As per ADNI protocols, all procedures performed in studies involving human participants were in accordance with the ethical standards of the Declaration of Helsinki. All participants provided written informed consent under these protocols. More details can be found at adni.loni.usc.edu.

## Supporting information



Supporting Information

Supporting Information
