# Supplementary material for: Benchmarking privacy and utility in synthetic tabular cohorts for Alzheimer's disease research
Source: Alzheimers Dement (Amst). 2026 Aug 10;18(3):e70430. doi: 10.1002/dad2.70430 (PMC13457355; doi:10.1002/dad2.70430)
Supplement: Supplementary file 1 [file DAD2-18-e70430-s001.pdf]

ICMJE DISCLOSURE FORM

Date: 1/22/2026

Your Name: Filip Winzell

Manuscript Title: Synthetic Alzheimer's disease dataset generation and evaluation with privacy protection

Manuscript Number (if known): DADM-D-25-00521

In the interest of transparency, we ask you to disclose all relationships/activities/interests listed below that are related to the content of your manuscript. "Related" means any relation with for-profit or not-for-profit third parties whose interests may be affected by the content of the manuscript. Disclosure represents a commitment to transparency and does not necessarily indicate a bias. If you are in doubt about whether to list a relationship/activity/interest, it is preferable that you do so.

The author's relationships/activities/interests should be defined broadly. For example, if your manuscript pertains to the epidemiology of hypertension, you should declare all relationships with manufacturers of antihypertensive medication, even if that medication is not mentioned in the manuscript.

In item #1 below, report all support for the work reported in this manuscript without time limit. For all other items, the time frame for disclosure is the past 36 months.

		**Name all entities with whom you have this relationship or indicate none (add rows as needed)**	**Specifications/Comments (e.g., if payments were made to you or to your institution)**
		**Time frame: Since the initial planning of the work**	
1	All support for the present manuscript (e.g., funding, provision of study materials, medical writing, article processing charges, etc.) No time limit for this item.	☐ None	
		WASP and DDLS	Grant to my institution
		National Institute of Aging	Grant/funding to my institution
		the Alzheimer's Association (and GHR foundation)	Click Grant/funding to my institution the tab key to add additional rows.
		the European Research Council	Grant/funding to my institution
		European Commission/Horizon Europe	Grant/funding to my institution
		ERA PerMed	Grant/funding to my institution
		The Michael J Fox Foundation	Grant/funding to my institution
		Global Research Platform LLC	Grant/funding to my institution
		the Cure Alzheimer's fund	Grant/funding to my institution
		Swedish Research Council	Grant/funding to my institution
		the Swedish Alzheimer Foundation	Grant/funding to my institution
		Swedish Brain Foundation	Grant/funding to my institution
		Strategic Research Area MultiPark	Grant/funding to my institution
		the Skåne University Hospital Foundation	Grant/funding to my institution
		Lilly Research Award Program	Grant/funding to my institution
		the Knut and Alice Wallenberg foundation	Grant/funding to my institution
		Konung Gustaf V:s och Drottning Victorias Frimurarestiftelse	Grant/funding to my institution
		the Kamprad Foundation	Grant/funding to my institution
		Bundy Academy, Greta and Johan Kock Foundation	Grant/funding to my institution
		the Crafoord Foundation	Grant/funding to my institution
		the Rönström Family Foundation	Grant/funding to my institution
		the Royal Physiographic Society	Grant/funding to my institution
		Stiftelsen för Gamla Tjänarinnor	Grant/funding to my institution

		Name all entities with whom you have this relationship or indicate none (add rows as needed)	Specifications/Comments (e.g., if payments were made to you or to your institution)
		Thorsten and Elsa Segerfalk's Foundation	Grant/funding to my institution
		Fredrik and Ingrid Thuring's Foundation	Grant/funding to my institution
Time frame: past 36 months			
**2**	Grants or contracts from any entity (if not indicated in item #1 above).	☒ **None**	
**3**	Royalties or licenses	☒ **None**	
**4**	Consulting fees	☒ **None**	
**5**	Payment or honoraria for lectures, presentations, speakers bureaus, manuscript writing or educational events	☒ **None**	
**6**	Payment for expert testimony	☒ **None**	
**7**	Support for attending meetings and/or travel	☒ **None**	

		Name all entities with whom you have this relationship or indicate none (add rows as needed)	Specifications/Comments (e.g., if payments were made to you or to your institution)
**8**	Patents planned, issued or pending	☒ **None**	
**9**	Participation on a Data Safety Monitoring Board or Advisory Board	☒ **None**	
**10**	Leadership or fiduciary role in other board, society, committee or advocacy group, paid or unpaid	☒ **None**	
**11**	Stock or stock options	☒ **None**	
**12**	Receipt of equipment, materials, drugs, medical writing, gifts or other services	☒ **None**	
**13**	Other financial or non-financial interests	☒ **None**	

Please place an "X" next to the following statement to indicate your agreement:

☒ I certify that I have answered every question and have not altered the wording of any of the questions on this form.

ICMJE DISCLOSURE FORM

Date: 1/29/2026

Your Name: Oskar Hansson

Manuscript Title: Synthetic Alzheimer's disease dataset generation and evaluation with privacy protection

Manuscript Number (if known): DADM-D-25-00521

In the interest of transparency, we ask you to disclose all relationships/activities/interests listed below that are related to the content of your manuscript. "Related" means any relation with for-profit or not-for-profit third parties whose interests may be affected by the content of the manuscript. Disclosure represents a commitment to transparency and does not necessarily indicate a bias. If you are in doubt about whether to list a relationship/activity/interest, it is preferable that you do so.

The author's relationships/activities/interests should be defined broadly. For example, if your manuscript pertains to the epidemiology of hypertension, you should declare all relationships with manufacturers of antihypertensive medication, even if that medication is not mentioned in the manuscript.

In item #1 below, report all support for the work reported in this manuscript without time limit. For all other items, the time frame for disclosure is the past 36 months.

		Name all entities with whom you have this relationship or indicate none (add rows as needed)	Specifications/Comments (e.g., if payments were made to you or to your institution)
Time frame: Since the initial planning of the work			
1	All support for the present manuscript (e.g., funding, provision of study materials, medical writing, article processing charges, etc.) **No time limit for this item.**	☒ None	
			Click the tab key to add additional rows.
Time frame: past 36 months			
2	Grants or contracts from any entity (if not indicated in item #1 above).	☐ **None**	
		Eli Lilly	I am an employee of Eli Lilly
3	Royalties or licenses	☒ **None**	

		Name all entities with whom you have this relationship or indicate none (add rows as needed)	Specifications/Comments (e.g., if payments were made to you or to your institution)
**4**	Consulting fees	☒ **None**	
**5**	Payment or honoraria for lectures, presentations, speakers bureaus, manuscript writing or educational events	☒ **None**	
**6**	Payment for expert testimony	☒ **None**	
**7**	Support for attending meetings and/or travel	☒ **None**	
**8**	Patents planned, issued or pending	☒ **None**	
**9**	Participation on a Data Safety Monitoring Board or Advisory Board	☒ **None**	
**10**	Leadership or fiduciary role in other board, society, committee or advocacy group, paid or unpaid	☒ **None**	

		Name all entities with whom you have this relationship or indicate none (add rows as needed)	Specifications/Comments (e.g., if payments were made to you or to your institution)
**11**	Stock or stock options	☒ **None**	
**12**	Receipt of equipment, materials, drugs, medical writing, gifts or other services	☒ **None**	
**13**	Other financial or non-financial interests	☒ **None**	

Please place an "X" next to the following statement to indicate your agreement:

☒ I certify that I have answered every question and have not altered the wording of any of the questions on this form.

ICMJE DISCLOSURE FORM

Date: 2/10/2026

Your Name: Niels Christian Overgaard

Manuscript Title: Synthetic Alzheimer's disease dataset generation and evaluation with privacy protection

Manuscript Number (if known): DADM-D-25-00521

In the interest of transparency, we ask you to disclose all relationships/activities/interests listed below that are related to the content of your manuscript. "Related" means any relation with for-profit or not-for-profit third parties whose interests may be affected by the content of the manuscript. Disclosure represents a commitment to transparency and does not necessarily indicate a bias. If you are in doubt about whether to list a relationship/activity/interest, it is preferable that you do so.

The author's relationships/activities/interests should be defined broadly. For example, if your manuscript pertains to the epidemiology of hypertension, you should declare all relationships with manufacturers of antihypertensive medication, even if that medication is not mentioned in the manuscript.

In item #1 below, report all support for the work reported in this manuscript without time limit. For all other items, the time frame for disclosure is the past 36 months.

		Name all entities with whom you have this relationship or indicate none (add rows as needed)	Specifications/Comments (e.g., if payments were made to you or to your institution)
		**Time frame: Since the initial planning of the work**	
1	All support for the present manuscript (e.g., funding, provision of study materials, medical writing, article processing charges, etc.) **No time limit for this item.**	☐ None	
		WASP and DDLS	Grant to my institution
		National Institute of Aging	Grant/funding to my institution
		the Alzheimer's Association (and GHR foundation)	Click Grant/funding to my institution the tab key to add additional rows.
		the European Research Council	Grant/funding to my institution
		European Commission/Horizon Europe	Grant/funding to my institution
		ERA PerMed	Grant/funding to my institution
		The Michael J Fox Foundation	Grant/funding to my institution
		Global Research Platform LLC	Grant/funding to my institution
		the Cure Alzheimer's fund	Grant/funding to my institution
		Swedish Research Council	Grant/funding to my institution
		the Swedish Alzheimer Foundation	Grant/funding to my institution
		Swedish Brain Foundation	Grant/funding to my institution
		Strategic Research Area MultiPark	Grant/funding to my institution
		the Skåne University Hospital Foundation	Grant/funding to my institution
		Lilly Research Award Program	Grant/funding to my institution
		the Knut and Alice Wallenberg foundation	Grant/funding to my institution
		Konung Gustaf V:s och Drottning Victorias Frimurarestiftelse	Grant/funding to my institution
		the Kamprad Foundation	Grant/funding to my institution
		Bundy Academy, Greta and Johan Kock Foundation	Grant/funding to my institution
		the Crafoord Foundation	Grant/funding to my institution
		the Rönström Family Foundation	Grant/funding to my institution
		the Royal Physiographic Society	Grant/funding to my institution
		Stiftelsen för Gamla Tjänarinnor	Grant/funding to my institution

		Name all entities with whom you have this relationship or indicate none (add rows as needed)	Specifications/Comments (e.g., if payments were made to you or to your institution)
		Thorsten and Elsa Segerfalk's Foundation	Grant/funding to my institution
		Fredrik and Ingrid Thuring's Foundation	Grant/funding to my institution
Time frame: past 36 months			
**2**	Grants or contracts from any entity (if not indicated in item #1 above).	☒ **None**	
**3**	Royalties or licenses	☒ **None**	
**4**	Consulting fees	☒ **None**	
**5**	Payment or honoraria for lectures, presentations, speakers bureaus, manuscript writing or educational events	☒ **None**	
**6**	Payment for expert testimony	☒ **None**	
**7**	Support for attending meetings and/or travel	☒ **None**	

		Name all entities with whom you have this relationship or indicate none (add rows as needed)	Specifications/Comments (e.g., if payments were made to you or to your institution)
**8**	Patents planned, issued or pending	☒ **None**	
**9**	Participation on a Data Safety Monitoring Board or Advisory Board	☒ **None**	
**10**	Leadership or fiduciary role in other board, society, committee or advocacy group, paid or unpaid	☒ **None**	
**11**	Stock or stock options	☒ **None**	
**12**	Receipt of equipment, materials, drugs, medical writing, gifts or other services	☒ **None**	
**13**	Other financial or non-financial interests	☒ **None**	

Please place an "X" next to the following statement to indicate your agreement:

☒ I certify that I have answered every question and have not altered the wording of any of the questions on this form.

ICMJE DISCLOSURE FORM

Date: 2/10/2026

Your Name: Jacob Vogel

Manuscript Title: Synthetic Alzheimer's disease dataset generation and evaluation with privacy protection

Manuscript Number (if known): DADM-D-25-00521

In the interest of transparency, we ask you to disclose all relationships/activities/interests listed below that are related to the content of your manuscript. "Related" means any relation with for-profit or not-for-profit third parties whose interests may be affected by the content of the manuscript. Disclosure represents a commitment to transparency and does not necessarily indicate a bias. If you are in doubt about whether to list a relationship/activity/interest, it is preferable that you do so.

The author's relationships/activities/interests should be defined broadly. For example, if your manuscript pertains to the epidemiology of hypertension, you should declare all relationships with manufacturers of antihypertensive medication, even if that medication is not mentioned in the manuscript.

In item #1 below, report all support for the work reported in this manuscript without time limit. For all other items, the time frame for disclosure is the past 36 months.

		Name all entities with whom you have this relationship or indicate none (add rows as needed)	Specifications/Comments (e.g., if payments were made to you or to your institution)
		**Time frame: Since the initial planning of the work**	
1	All support for the present manuscript (e.g., funding, provision of study materials, medical writing, article processing charges, etc.) No time limit for this item.	☐ None	
		WASP and DDLS	Grant to my institution
		National Institute of Aging	Grant/funding to my institution
		the Alzheimer's Association (and GHR foundation)	Click Grant/funding to my institution the tab key to add additional rows.
		the European Research Council	Grant/funding to my institution
		European Commission/Horizon Europe	Grant/funding to my institution
		ERA PerMed	Grant/funding to my institution
		The Michael J Fox Foundation	Grant/funding to my institution
		Global Research Platform LLC	Grant/funding to my institution
		the Cure Alzheimer's fund	Grant/funding to my institution
		Swedish Research Council	Grant/funding to my institution
		the Swedish Alzheimer Foundation	Grant/funding to my institution
		Swedish Brain Foundation	Grant/funding to my institution
		Strategic Research Area MultiPark	Grant/funding to my institution
		the Skåne University Hospital Foundation	Grant/funding to my institution
		Lilly Research Award Program	Grant/funding to my institution
		the Knut and Alice Wallenberg foundation	Grant/funding to my institution
		Konung Gustaf V:s och Drottning Victorias Frimurarestiftelse	Grant/funding to my institution
		the Kamprad Foundation	Grant/funding to my institution
		Bundy Academy, Greta and Johan Kock Foundation	Grant/funding to my institution
		the Crafoord Foundation	Grant/funding to my institution
		the Rönström Family Foundation	Grant/funding to my institution
		the Royal Physiographic Society	Grant/funding to my institution
		Stiftelsen för Gamla Tjänarinnor	Grant/funding to my institution

		Name all entities with whom you have this relationship or indicate none (add rows as needed)	Specifications/Comments (e.g., if payments were made to you or to your institution)
		Thorsten and Elsa Segerfalk's Foundation	Grant/funding to my institution
		Fredrik and Ingrid Thuring's Foundation	Grant/funding to my institution
		**Time frame: past 36 months**	
**2**	Grants or contracts from any entity (if not indicated in item #1 above).	☒ **None**	
**3**	Royalties or licenses	☒ **None**	
**4**	Consulting fees	☒ **None**	
**5**	Payment or honoraria for lectures, presentations, speakers bureaus, manuscript writing or educational events	☒ **None**	
**6**	Payment for expert testimony	☒ **None**	
**7**	Support for attending meetings and/or travel	☒ **None**	

		Name all entities with whom you have this relationship or indicate none (add rows as needed)	Specifications/Comments (e.g., if payments were made to you or to your institution)
**8**	Patents planned, issued or pending	☒ **None**	
**9**	Participation on a Data Safety Monitoring Board or Advisory Board	☒ **None**	
**10**	Leadership or fiduciary role in other board, society, committee or advocacy group, paid or unpaid	☒ **None**	
**11**	Stock or stock options	☒ **None**	
**12**	Receipt of equipment, materials, drugs, medical writing, gifts or other services	☒ **None**	
**13**	Other financial or non-financial interests	☒ **None**	

Please place an "X" next to the following statement to indicate your agreement:

☒ I certify that I have answered every question and have not altered the wording of any of the questions on this form.

ICMJE DISCLOSURE FORM

**Date:**	1/29/2026
**Your Name:**	Karl (Kalle) Åström
**Manuscript Title:**	Synthetic Alzheimer's disease dataset generation and evaluation with privacy protection
**Manuscript Number (if known):**	DADM-D-25-00521

In the interest of transparency, we ask you to disclose all relationships/activities/interests listed below that are related to the content of your manuscript. "Related" means any relation with for-profit or not-for-profit third parties whose interests may be affected by the content of the manuscript. Disclosure represents a commitment to transparency and does not necessarily indicate a bias. If you are in doubt about whether to list a relationship/activity/interest, it is preferable that you do so.

The author's relationships/activities/interests should be defined broadly. For example, if your manuscript pertains to the epidemiology of hypertension, you should declare all relationships with manufacturers of antihypertensive medication, even if that medication is not mentioned in the manuscript.

In item #1 below, report all support for the work reported in this manuscript without time limit. For all other items, the time frame for disclosure is the past 36 months.

		**Name all entities with whom you have this relationship or indicate none (add rows as needed)**	**Specifications/Comments (e.g., if payments were made to you or to your institution)**
		**Time frame: Since the initial planning of the work**	
1	All support for the present manuscript (e.g., funding, provision of study materials, medical writing, article processing charges, etc.) **No time limit for this item.**	☐ None	
		WASP and DDLS	Grant to my institution
		National Institute of Aging	Grant/funding to my institution
		the Alzheimer's Association (and GHR foundation)	Click Grant/funding to my institution the tab key to add additional rows.
		the European Research Council	Grant/funding to my institution
		European Commission/Horizon Europe	Grant/funding to my institution
		ERA PerMed	Grant/funding to my institution
		The Michael J Fox Foundation	Grant/funding to my institution
		Global Research Platform LLC	Grant/funding to my institution
		the Cure Alzheimer's fund	Grant/funding to my institution
		Swedish Research Council	Grant/funding to my institution
		the Swedish Alzheimer Foundation	Grant/funding to my institution
		Swedish Brain Foundation	Grant/funding to my institution
		Strategic Research Area MultiPark	Grant/funding to my institution
		the Skåne University Hospital Foundation	Grant/funding to my institution
		Lilly Research Award Program	Grant/funding to my institution
		the Knut and Alice Wallenberg foundation	Grant/funding to my institution
		Konung Gustaf V:s och Drottning Victorias Frimurarestiftelse	Grant/funding to my institution
		the Kamprad Foundation	Grant/funding to my institution
		Bundy Academy, Greta and Johan Kock Foundation	Grant/funding to my institution
		the Crafoord Foundation	Grant/funding to my institution
		the Rönström Family Foundation	Grant/funding to my institution
		the Royal Physiographic Society	Grant/funding to my institution
		Stiftelsen för Gamla Tjänarinnor	Grant/funding to my institution

		Name all entities with whom you have this relationship or indicate none (add rows as needed)	Specifications/Comments (e.g., if payments were made to you or to your institution)
		Thorsten and Elsa Segerfalk's Foundation	Grant/funding to my institution
		Fredrik and Ingrid Thuring's Foundation	Grant/funding to my institution
**Time frame: past 36 months**			
**2**	Grants or contracts from any entity (if not indicated in item #1 above).	☒ **None**	
**3**	Royalties or licenses	☒ **None**	
**4**	Consulting fees	☒ **None**	
**5**	Payment or honoraria for lectures, presentations, speakers bureaus, manuscript writing or educational events	☒ **None**	
**6**	Payment for expert testimony	☒ **None**	
**7**	Support for attending meetings and/or travel	☒ **None**	

		Name all entities with whom you have this relationship or indicate none (add rows as needed)	Specifications/Comments (e.g., if payments were made to you or to your institution)
**8**	Patents planned, issued or pending	☒ **None**	
**9**	Participation on a Data Safety Monitoring Board or Advisory Board	☒ **None**	
**10**	Leadership or fiduciary role in other board, society, committee or advocacy group, paid or unpaid	☒ **None**	
**11**	Stock or stock options	☐ **None**	
		Kroton AB	
		Inceptron AB	Startup company – hardware acceleration for AI
**12**	Receipt of equipment, materials, drugs, medical writing, gifts or other services	☒ **None**	
**13**	Other financial or non-financial interests	☒ **None**	

Please place an "X" next to the following statement to indicate your agreement:

☒ I certify that I have answered every question and have not altered the wording of any of the questions on this form.

ICMJE DISCLOSURE FORM

**Date:**	1/29/2026
**Your Name:**	Anders Heyden
**Manuscript Title:**	Synthetic Alzheimer's disease dataset generation and evaluation with privacy protection
**Manuscript Number (if known):**	DADM-D-25-00521

In the interest of transparency, we ask you to disclose all relationships/activities/interests listed below that are related to the content of your manuscript. "Related" means any relation with for-profit or not-for-profit third parties whose interests may be affected by the content of the manuscript. Disclosure represents a commitment to transparency and does not necessarily indicate a bias. If you are in doubt about whether to list a relationship/activity/interest, it is preferable that you do so.

The author's relationships/activities/interests should be defined broadly. For example, if your manuscript pertains to the epidemiology of hypertension, you should declare all relationships with manufacturers of antihypertensive medication, even if that medication is not mentioned in the manuscript.

In item #1 below, report all support for the work reported in this manuscript without time limit. For all other items, the time frame for disclosure is the past 36 months.

		**Name all entities with whom you have this relationship or indicate none (add rows as needed)**	**Specifications/Comments (e.g., if payments were made to you or to your institution)**
		Time frame: Since the initial planning of the work	
1	All support for the present manuscript (e.g., funding, provision of study materials, medical writing, article processing charges, etc.) No time limit for this item.	☒ None	

		Name all entities with whom you have this relationship or indicate none (add rows as needed)	Specifications/Comments (e.g., if payments were made to you or to your institution)
Time frame: past 36 months			
**2**	Grants or contracts from any entity (if not indicated in item #1 above).	☒ **None**	
**3**	Royalties or licenses	☒ **None**	
**4**	Consulting fees	☒ **None**	
**5**	Payment or honoraria for lectures, presentations, speakers bureaus, manuscript writing or educational events	☒ **None**	
**6**	Payment for expert testimony	☒ **None**	
**7**	Support for attending meetings and/or travel	☒ **None**	
**8**	Patents planned, issued or pending	☒ **None**	

		Name all entities with whom you have this relationship or indicate none (add rows as needed)	Specifications/Comments (e.g., if payments were made to you or to your institution)
**9**	Participation on a Data Safety Monitoring Board or Advisory Board	☒ **None**	
**10**	Leadership or fiduciary role in other board, society, committee or advocacy group, paid or unpaid	☒ **None**	
**11**	Stock or stock options	☒ **None**	
**12**	Receipt of equipment, materials, drugs, medical writing, gifts or other services	☒ **None**	
**13**	Other financial or non-financial interests	☒ **None**	

Please place an "X" next to the following statement to indicate your agreement:

☒ I certify that I have answered every question and have not altered the wording of any of the questions on this form.

ICMJE DISCLOSURE FORM

Date: 2/8/2026

Your Name: Linda Karlsson

Manuscript Title: Synthetic Alzheimer's disease dataset generation and evaluation with privacy protection

Manuscript Number (if known): DADM-D-25-00521

In the interest of transparency, we ask you to disclose all relationships/activities/interests listed below that are related to the content of your manuscript. "Related" means any relation with for-profit or not-for-profit third parties whose interests may be affected by the content of the manuscript. Disclosure represents a commitment to transparency and does not necessarily indicate a bias. If you are in doubt about whether to list a relationship/activity/interest, it is preferable that you do so.

The author's relationships/activities/interests should be defined broadly. For example, if your manuscript pertains to the epidemiology of hypertension, you should declare all relationships with manufacturers of antihypertensive medication, even if that medication is not mentioned in the manuscript.

In item #1 below, report all support for the work reported in this manuscript without time limit. For all other items, the time frame for disclosure is the past 36 months.

		**Name all entities with whom you have this relationship or indicate none (add rows as needed)**	**Specifications/Comments (e.g., if payments were made to you or to your institution)**
		Time frame: Since the initial planning of the work	
1	All support for the present manuscript (e.g., funding, provision of study materials, medical writing, article processing charges, etc.) **No time limit for this item.**	☐ None	
		WASP and DDLS	Grant to my institution
		National Institute of Aging	Grant/funding to my institution
		the Alzheimer's Association (and GHR foundation)	Click Grant/funding to my institution the tab key to add additional rows.
		the European Research Council	Grant/funding to my institution
		European Commission/Horizon Europe	Grant/funding to my institution
		ERA PerMed	Grant/funding to my institution
		The Michael J Fox Foundation	Grant/funding to my institution
		Global Research Platform LLC	Grant/funding to my institution
		the Cure Alzheimer's fund	Grant/funding to my institution
		Swedish Research Council	Grant/funding to my institution
		the Swedish Alzheimer Foundation	Grant/funding to my institution
		Swedish Brain Foundation	Grant/funding to my institution
		Strategic Research Area MultiPark	Grant/funding to my institution
		the Skåne University Hospital Foundation	Grant/funding to my institution
		Lilly Research Award Program	Grant/funding to my institution
		the Knut and Alice Wallenberg foundation	Grant/funding to my institution
		Konung Gustaf V:s och Drottning Victorias Frimurarestiftelse	Grant/funding to my institution
		the Kamprad Foundation	Grant/funding to my institution
		Bundy Academy, Greta and Johan Kock Foundation	Grant/funding to my institution
		the Crafoord Foundation	Grant/funding to my institution
		the Rönström Family Foundation	Grant/funding to my institution
		the Royal Physiographic Society	Grant/funding to my institution
		Stiftelsen för Gamla Tjänarinnor	Grant/funding to my institution

		Name all entities with whom you have this relationship or indicate none (add rows as needed)	Specifications/Comments (e.g., if payments were made to you or to your institution)
		Thorsten and Elsa Segerfalk's Foundation	Grant/funding to my institution
		Fredrik and Ingrid Thuring's Foundation	Grant/funding to my institution
		**Time frame: past 36 months**	
**2**	Grants or contracts from any entity (if not indicated in item #1 above).	☒ **None**	
**3**	Royalties or licenses	☒ **None**	
**4**	Consulting fees	☒ **None**	
**5**	Payment or honoraria for lectures, presentations, speakers bureaus, manuscript writing or educational events	☒ **None**	
**6**	Payment for expert testimony	☒ **None**	
**7**	Support for attending meetings and/or travel	☒ **None**	

		Name all entities with whom you have this relationship or indicate none (add rows as needed)	Specifications/Comments (e.g., if payments were made to you or to your institution)
**8**	Patents planned, issued or pending	☒ **None**	
**9**	Participation on a Data Safety Monitoring Board or Advisory Board	☒ **None**	
**10**	Leadership or fiduciary role in other board, society, committee or advocacy group, paid or unpaid	☒ **None**	
**11**	Stock or stock options	☒ **None**	
**12**	Receipt of equipment, materials, drugs, medical writing, gifts or other services	☒ **None**	
**13**	Other financial or non-financial interests	☒ **None**	

Please place an "X" next to the following statement to indicate your agreement:

☒ I certify that I have answered every question and have not altered the wording of any of the questions on this form.

ICMJE DISCLOSURE FORM

**Date:**	1/29/2026
**Your Name:**	Niklas Mattsson-Carlgren
**Manuscript Title:**	Synthetic Alzheimer's disease dataset generation and evaluation with privacy protection
**Manuscript Number (if known):**	DADM-D-25-00521

In the interest of transparency, we ask you to disclose all relationships/activities/interests listed below that are related to the content of your manuscript. "Related" means any relation with for-profit or not-for-profit third parties whose interests may be affected by the content of the manuscript. Disclosure represents a commitment to transparency and does not necessarily indicate a bias. If you are in doubt about whether to list a relationship/activity/interest, it is preferable that you do so.

The author's relationships/activities/interests should be defined broadly. For example, if your manuscript pertains to the epidemiology of hypertension, you should declare all relationships with manufacturers of antihypertensive medication, even if that medication is not mentioned in the manuscript.

In item #1 below, report all support for the work reported in this manuscript without time limit. For all other items, the time frame for disclosure is the past 36 months.

		**Name all entities with whom you have this relationship or indicate none (add rows as needed)**	**Specifications/Comments (e.g., if payments were made to you or to your institution)**
		Time frame: Since the initial planning of the work	
1	All support for the present manuscript (e.g., funding, provision of study materials, medical writing, article processing charges, etc.) **No time limit for this item.**	☐ None	
		WASP and DDLS	Grant to my institution
		the Alzheimer's Association (and GHR foundation)	Click Grant/funding to my institution the tab key to add additional rows.
		ERA PerMed	Grant/funding to my institution
		The Michael J Fox Foundation	Grant/funding to my institution
		Global Research Platform LLC	Grant/funding to my institution
		the Cure Alzheimer's fund	Grant/funding to my institution
		Swedish Research Council	Grant/funding to my institution
		the Swedish Alzheimer Foundation	Grant/funding to my institution
		Swedish Brain Foundation	Grant/funding to my institution
		Strategic Research Area MultiPark	Grant/funding to my institution
		the Skåne University Hospital Foundation	Grant/funding to my institution
		Lilly Research Award Program	Grant/funding to my institution
		the Knut and Alice Wallenberg foundation	Grant/funding to my institution
		Konung Gustaf V:s och Drottning Victorias Frimurarestiftelse	Grant/funding to my institution
		Bundy Academy, Greta and Johan Kock Foundation	Grant/funding to my institution
		the Rönström Family Foundation	Grant/funding to my institution
		Stiftelsen för Gamla Tjänarinnor	Grant/funding to my institution
		Time frame: past 36 months	

		Name all entities with whom you have this relationship or indicate none (add rows as needed)	Specifications/Comments (e.g., if payments were made to you or to your institution)
**2**	Grants or contracts from any entity (if not indicated in item #1 above).	☒ **None**	
**3**	Royalties or licenses	☒ **None**	
**4**	Consulting fees	☐ **None**	
		Biogen	Merck
		BioArctic	Owkin
		Eli Lilly	Novo Nordisk
		Roche	
**5**	Payment or honoraria for lectures, presentations, speakers bureaus, manuscript writing or educational events	☒ **None**	
**6**	Payment for expert testimony	☒ **None**	
**7**	Support for attending meetings and/or travel	☒ **None**	
**8**	Patents planned, issued or pending	☒ **None**	
**9**	Participation on a Data Safety	☒ **None**	

		Name all entities with whom you have this relationship or indicate none (add rows as needed)	Specifications/Comments (e.g., if payments were made to you or to your institution)
	Monitoring Board or Advisory Board		
**10**	Leadership or fiduciary role in other board, society, committee or advocacy group, paid or unpaid	☒ **None**	
**11**	Stock or stock options	☒ **None**	
**12**	Receipt of equipment, materials, drugs, medical writing, gifts or other services	☒ **None**	
**13**	Other financial or non-financial interests	☒ **None**	

Please place an "X" next to the following statement to indicate your agreement:

☒ I certify that I have answered every question and have not altered the wording of any of the questions on this form.

ICMJE DISCLOSURE FORM

Date: 1/29/2026

Your Name: Ida Arvidsson

Manuscript Title: Synthetic Alzheimer's disease dataset generation and evaluation with privacy protection

Manuscript Number (if known): DADM-D-25-00521

In the interest of transparency, we ask you to disclose all relationships/activities/interests listed below that are related to the content of your manuscript. "Related" means any relation with for-profit or not-for-profit third parties whose interests may be affected by the content of the manuscript. Disclosure represents a commitment to transparency and does not necessarily indicate a bias. If you are in doubt about whether to list a relationship/activity/interest, it is preferable that you do so.

The author's relationships/activities/interests should be defined broadly. For example, if your manuscript pertains to the epidemiology of hypertension, you should declare all relationships with manufacturers of antihypertensive medication, even if that medication is not mentioned in the manuscript.

In item #1 below, report all support for the work reported in this manuscript without time limit. For all other items, the time frame for disclosure is the past 36 months.

		Name all entities with whom you have this relationship or indicate none (add rows as needed)	Specifications/Comments (e.g., if payments were made to you or to your institution)
Time frame: Since the initial planning of the work			
1	All support for the present manuscript (e.g., funding, provision of study materials, medical writing, article processing charges, etc.) No time limit for this item.	☐ None	
		WASP and DDLS	Grant to my institution
		National Institute of Aging	Grant/funding to my institution
		the Alzheimer's Association (and GHR foundation)	Click Grant/funding to my institution the tab key to add additional rows.
		the European Research Council	Grant/funding to my institution
		European Commission/Horizon Europe	Grant/funding to my institution
		ERA PerMed	Grant/funding to my institution
		The Michael J Fox Foundation	Grant/funding to my institution
		Global Research Platform LLC	Grant/funding to my institution
		the Cure Alzheimer's fund	Grant/funding to my institution
		Swedish Research Council	Grant/funding to my institution
		the Swedish Alzheimer Foundation	Grant/funding to my institution
		Swedish Brain Foundation	Grant/funding to my institution
		Strategic Research Area MultiPark	Grant/funding to my institution
		the Skåne University Hospital Foundation	Grant/funding to my institution
		Lilly Research Award Program	Grant/funding to my institution
		the Knut and Alice Wallenberg foundation	Grant/funding to my institution
		Konung Gustaf V:s och Drottning Victorias Frimurarestiftelse	Grant/funding to my institution
		the Kamprad Foundation	Grant/funding to my institution
		Bundy Academy, Greta and Johan Kock Foundation	Grant/funding to my institution
		the Crafoord Foundation	Grant/funding to my institution
		the Rönström Family Foundation	Grant/funding to my institution
		the Royal Physiographic Society	Grant/funding to my institution
		Stiftelsen för Gamla Tjänarinnor	Grant/funding to my institution

		Name all entities with whom you have this relationship or indicate none (add rows as needed)	Specifications/Comments (e.g., if payments were made to you or to your institution)
		Thorsten and Elsa Segerfalk's Foundation	Grant/funding to my institution
		Fredrik and Ingrid Thuring's Foundation	Grant/funding to my institution
		**Time frame: past 36 months**	
**2**	Grants or contracts from any entity (if not indicated in item #1 above).	☒ **None**	
**3**	Royalties or licenses	☒ **None**	
**4**	Consulting fees	☒ **None**	
**5**	Payment or honoraria for lectures, presentations, speakers bureaus, manuscript writing or educational events	☒ **None**	
**6**	Payment for expert testimony	☒ **None**	
**7**	Support for attending meetings and/or travel	☒ **None**	

		Name all entities with whom you have this relationship or indicate none (add rows as needed)	Specifications/Comments (e.g., if payments were made to you or to your institution)
**8**	Patents planned, issued or pending	☒ **None**	
**9**	Participation on a Data Safety Monitoring Board or Advisory Board	☒ **None**	
**10**	Leadership or fiduciary role in other board, society, committee or advocacy group, paid or unpaid	☒ **None**	
**11**	Stock or stock options	☒ **None**	
**12**	Receipt of equipment, materials, drugs, medical writing, gifts or other services	☒ **None**	
**13**	Other financial or non-financial interests	☒ **None**	

Please place an "X" next to the following statement to indicate your agreement:

☒ I certify that I have answered every question and have not altered the wording of any of the questions on this form.

ICMJE DISCLOSURE FORM

Date: 2/10/2026

Your Name: Alzheimer's Disease Neuroimaging Initiative Alzheimer's Disease Neuroimaging Initiative

Manuscript Title: Synthetic Alzheimer's disease dataset generation and evaluation with privacy protection

Manuscript Number (if known): DADM-D-25-00521

In the interest of transparency, we ask you to disclose all relationships/activities/interests listed below that are related to the content of your manuscript. "Related" means any relation with for-profit or not-for-profit third parties whose interests may be affected by the content of the manuscript. Disclosure represents a commitment to transparency and does not necessarily indicate a bias. If you are in doubt about whether to list a relationship/activity/interest, it is preferable that you do so.

The author's relationships/activities/interests should be defined broadly. For example, if your manuscript pertains to the epidemiology of hypertension, you should declare all relationships with manufacturers of antihypertensive medication, even if that medication is not mentioned in the manuscript.

In item #1 below, report all support for the work reported in this manuscript without time limit. For all other items, the time frame for disclosure is the past 36 months.

		Name all entities with whom you have this relationship or indicate none (add rows as needed)	Specifications/Comments (e.g., if payments were made to you or to your institution)
		**Time frame: Since the initial planning of the work**	
1	All support for the present manuscript (e.g., funding, provision of study materials, medical writing, article processing charges, etc.) **No time limit for this item.**	☒ None	
			Click the tab key to add additional rows.
		**Time frame: past 36 months**	
2	Grants or contracts from any entity (if not indicated in item #1 above).	☒ **None**	
3	Royalties or licenses	☒ **None**	

		Name all entities with whom you have this relationship or indicate none (add rows as needed)	Specifications/Comments (e.g., if payments were made to you or to your institution)
**4**	Consulting fees	☒ **None**	
**5**	Payment or honoraria for lectures, presentations, speakers bureaus, manuscript writing or educational events	☒ **None**	
**6**	Payment for expert testimony	☒ **None**	
**7**	Support for attending meetings and/or travel	☒ **None**	
**8**	Patents planned, issued or pending	☒ **None**	
**9**	Participation on a Data Safety Monitoring Board or Advisory Board	☒ **None**	
**10**	Leadership or fiduciary role in other board, society, committee or advocacy group, paid or unpaid	☒ **None**	

		Name all entities with whom you have this relationship or indicate none (add rows as needed)	Specifications/Comments (e.g., if payments were made to you or to your institution)
**11**	Stock or stock options	☒ **None**	
**12**	Receipt of equipment, materials, drugs, medical writing, gifts or other services	☒ **None**	
**13**	Other financial or non-financial interests	☒ **None**	

Please place an "X" next to the following statement to indicate your agreement:

☒ I certify that I have answered every question and have not altered the wording of any of the questions on this form.
