# Supplementary material for: Benchmarking privacy and utility in synthetic tabular cohorts for Alzheimer's disease research
Source: Alzheimers Dement (Amst). 2026 Aug 10;18(3):e70430. doi: 10.1002/dad2.70430 (PMC13457355; doi:10.1002/dad2.70430)
Supplement: Supplementary file 2 [file DAD2-18-e70430-s002.pdf]

Benchmarking privacy and utility in synthetic tabular cohorts for Alzheimer's disease research

Appendix A. Supplementary material

Appendix A.1. Dataset summary

The datasets from ADNI and the A4 study are summarized in Table A.1. In the ADNI+ cohort we included the volumetric MRI measurements summarized in Table A.2

Appendix A.2. Pre-processing

Before any analysis, we investigated possible outliers in the data. For the MRI variables, we observed a few suspicious measurements with abnormally large regional volumes. Thus, we proceeded by identifying outliers by converting them to z-scores. Since some of the MRI variables might not be normally distributed, we also calculated z-scores after applying a natural logarithm transformation. Given the size of the dataset, any observations with larger z-scores than 3.89 should be unlikely, if they are normally distributed. Hence we used a threshold of 4 (corresponding with a confidence level of roughly 99.99%) and classified observations as outiers based on the original or log-transformed values given which produced the fewest number. For the other variables, such as biomarkers and cognitive tests, we hypothesized that the intrinsic uncertainty of the diagnostic procedure might cause any outliers. Since capturing this uncertainty is desirable when generating synthetic data, we kept the original data points without any outlier removal.

Appendix A.3. Greedy Bayes

DataSynthesizer (DS) [1] uses the GreedyBayes algorithm [2] to generate synthetic tabular data. It takes an input dataset D, with n tuples of a set of d attributes (A) and one hyperparameter defining the maximum number of parent nodes k (referred to as the degree of the network). The algorithm begins by randomly selecting a root (X_1) from A and adding the attribute-parent pair (X_1, ϕ) to the network (i.e. the parent set of the root is the empty set, ϕ). Then for $i = 2, \ldots, d$, attribute-parent set pairs (X_i, Π_i) are added iteratively by greedily choosing the pair that maximizes the mutual information, defined as

$$I(X, \Pi) = \sum_{x \in X} \sum_{\pi \in \Pi} P(x, \pi) \log_2 \Big(\frac{P(x, \pi)}{P(x)P(\pi)} \Big), \tag{A.1}$$

where Π_i is the parent set, $P(\cdot)$ denotes the marginal probability, and $P(\cdot, \cdot)$ denotes the joint probability. The attribute-parent pairs available for the algorithm to choose from are limited such that the number of parent nodes is maximum k ($|\Pi_i| \leq k$) and to ensure that N remains a directed acyclic graph (DAG). During generation, the root is sampled from an unconditioned probability

Table A.1: Summary of the training and test datasets for both the ADNI and A4 datasets. For numerical variables, the mean and the standard deviation are presented, and for categorical variables, the distribution in percent. The missing value ratio for each attribute in the respective dataset is also presented. Note that for volumetric MRI, the Left and Right regions are reported in A4, while the total volume is used in ADNI.

**Variable**	**ADNI**				**A4**			
	Train (n = 1925)	Missing [%]	Test (n = 482)	Missing [%]	Train (n = 3593)	Missing [%]	Test (n = 899)	Missing [%]
**Demographics**								
Age (years)	72.87 ± 7.46	0.1	73.07 ± 6.96	0.2	71.28 ± 4.65	0	71.34 ± 4.76	0
Sex (M/F) [%]	52.5 / 47.5	0	52.3 / 47.7	0	41.1 / 58.8	0	38.4 / 61.5	0
Education (years)	16.0 ± 2.7	0	16.0 ± 2.8	0	16.6 ± 2.8	0.1	16.6 ± 2.8	0.3
APOE ϵ4 (0/1/2) [%]	49.3 / 33.6 / 9.0	8.1	51.2 / 32.3 / 7.5	8.9				
Ethnicity (Hispanic, Not Hisp.)					3.0 / 96.1	0.8	3.5 / 95.8	0.6
Marital status (Married / Wid. / Div. / Not Mar.)					70.2 / 9.6 / 14.2 / 4.2	1.8	72.2 / 9.1 / 13.0 / 3.6	2.0
Retired (Yes/No)					22.6 / 76.0	1.3	23.5 / 75.1	1.4
Residence (1–6, 1: Indep., 2–6 assisted/others)					98.0 / 1.2 / 0.5 / 0.5 / 0 / 0.3	0	98.3 / 1.0 / 0.3 0 / 0 / 0.2	0
**CSF biomarkers**								
Aβ_{42} [pg/mL]	1049.31 ± 606.39	48.7	1071 ± 598	52.9				
P-TAU [pg/mL]	27.89 ± 14.38	48.9	26.6 ± 14.1	53.3				
TAU [pg/mL]	289.03 ± 129.39	48.9	277.4 ± 128.8	53.3				
**Imaging**								
Amyloid-β PET (AV45)	1.21 ± 0.23	53.2	1.19 ± 0.22	52.7	1.11 ± 0.17	0	1.10 ± 0.17	0
Hippocampus (Tot. / Left) [mm^3]	6924 ± 1181	15.6	6914 ± 1164	15.3	3313 ± 421	71.6	3302 ± 380	73.2
(Right) [mm^3]					3435 ± 437	71.6	3426 ± 394	73.2
Entorhinal (Tot. / Left) [mm^3]	3625 ± 823	16.9	3630 ± 799	16.2	2826 ± 605	71.6	2752 ± 518	73.2
(Right) [mm^3]					2589 ± 582	71.6	2600 ± 606	73.2
Fusiform (Tot. / Left) [mm^3]	17598 ± 2757	16.9	17720 ± 2763	16.2	10168 ± 1209	71.6	10176 ± 1182	73.2
(Right) [mm^3]					12088 ± 1468	71.6	12045 ± 1492	73.2
Cortical GM (Left) [mm^3]					3159 ± 518	71.6	3154 ± 594	73.2
Cortical GM (Right) [mm^3]					2756 ± 392	71.6	2762 ± 435	73.2
Ventricles [mm^3]	39091 ± 22396	8.1	40324 ± 21831	7.9				
WholeBrain [mm^3]	$1.03 \pm 0.11 \cdot 10^6$	6.8	$1.03 \pm 0.11 \cdot 10^6$	6.6				
MidTemp [mm^3]	19753 ± 3076	17.0	19860 ± 3176	16.2				
ICV [mm^3]	$1.51 \pm 0.17 \cdot 10^6$	5.4	$1.52 \pm 0.17 \cdot 10^6$	4.8				
**Assessments**								
ADAS13	15.85 ± 9.48	0.8	15.79 ± 9.69	0.6				
MMSE	27.38 ± 2.64		27.38 ± 2.69	0	28.78 ± 1.23	0.1	28.85 ± 1.19	0.0
Logical Mem. (Immediate)					12.9 ± 3.2	0	12.9 ± 3.1	0
Logical Mem. (Delayed)					11.7 ± 3.2	0	11.7 ± 3.2	0
C-Path Q.					22.7 ± 11.3	4.0	22.7 ± 10.8	3.5
Digit Symbol					43.6 ± 9.0	0	44.2 ± 8.8	0
CDR-SOB					0.06 ± 0.16	0	0.06 ± 0.18	0
**Diagnostic labels**								
Diagnosis (CN/MCI/AD) [%]	37.0 / 45.6 / 17.5	0	37.0 / 46.5 / 16.0	0				
AB status (-/+) [%]					69.9 / 30.0	0	72.4 / 27.5	0

Table A.2: MRI volumetric variables used in the ADNI+ cohort.

**Region**	**Variable codes** (Right/Left)	
Insula	ST130CV	ST129CV
Transverse Temporal	ST121CV	ST62CV
Temporal Pole	ST119CV	ST60CV
Superior Temporal	ST117CV	ST58CV
Superior Parietal	ST116CV	ST57CV
Superior Frontal	ST115CV	ST56CV
Precuneus	ST111CV	ST52CV
Middle Temporal	ST99CV	ST40CV
Lingual	ST97CV	ST38CV
Lateral Ventricle	ST96SV	ST37SV
Inferior Temporal	ST91CV	ST32CV
Inferior Parietal	ST90CV	ST31CV
Inferior Lateral Ventricle	ST89SV	ST30SV
Hippocampus	ST88SV	ST29SV
Fusiform	ST85CV	ST26CV
Entorhinal	ST83CV	ST24CV
Cerebellum White Matter	ST77SV	ST18SV
Third Ventricle	ST127SV	

distribution, then the attributes are sampled by order of the constructed network N, using noisy conditioned distributions. The noise, which is sampled from a Laplace distribution ($Lap(\frac{4(d-k)}{n\epsilon})$), is injected to preserve privacy.

Appendix A.4. SynthEval metrics

One of SynthEval's most noteworthy contributions is the nearest neighbor (NN) module, which is used to find the closest data points both within and between the datasets. It uses Gower's distance measure instead of Euclidean, which enables a normalized distance measure of both categorical and numerical variables combined in this high-dimensional space. For example, this is used in calculating the nearest neighbor adversarial accuracy (NNAA) and the nearest neighbor distance ratio (NNDR). The NNAA can be seen as a theoretical measure of how well an adversarial classifier could differentiate between the real and synthetic data[3]. Given real and synthetic datasets, it is defined as

$$\text{NNAA} = \frac{1}{2}\Big(\frac{1}{N}\sum_{i}^{N}\mathbb{1}(d_{RS}(i) > d_{RR}(i)) + \frac{1}{N}\sum_{i}^{N}\mathbb{1}(d_{SR}(i) > d_{SS}(i))\Big), \tag{A.2}$$

where $d_{RS}(i)$ is the NN distance from subject i in the real dataset to the synthetic (and $d_{SR}(i)$ the opposite). The $d_{RR}(i)$ and $d_{SS}(i)$ are the "leave-one-out" NN distances within the real and synthetic datasets respectively. The $\mathbb{1}$ function takes the value one if the argument is true and zero otherwise[4]. Hence, a value close to one indicates that the real and synthetic are completely separated. However, a value close to zero implies overfitting and increases the risk of privacy

violation. The optimum is somewhat ambiguous, and therefore the privacy loss is a more meaningful metric[5], which is defined as the difference in metric evaluated on the training dataset and the hold-out test dataset[3].

Hitting rate is simply finding any exact matches from the real dataset in the synthetic. For numerical variables to be considered exact, a threshold of 1/30th of the variable range is used. ϵ-identifiability is the ratio of real samples that are closer (or not far enough) to a synthetic than another real. Hence, for an ϵ-identifiable synthetic dataset, the probability that a real record is too similar to a synthetic is less than ϵ. $\epsilon = 0$ means that the datasets are perfectly non-identifiable and $\epsilon = 1$ the opposite[6]. The MIA metric simulates a membership inference attack, where an adversary has obtained a large dataset which contains the real patients used for synthetic data generation, and aims to identify them. It uses a random forest classifier trained to distinguish the synthetic dataset from an external one, and the recall of the model in identifying training samples in a mix of training and hold-out test samples indicates privacy risks[3]. Finally, the ADR is also based on an adversarial scenario, where an adversary wants to use the synthetic dataset to infer missing attributes of the real. In SynthEval this is implemented as the worst-case scenario, where the adversary has access to all attributes except one. The average accuracy of a predictive model is reported as the risk. Similarly, as with the Hitting rate, a threshold is used for the numerical attributes to decide whether the predictions are correct[3]. These metrics reports recall values between 0-1, where lower values indicate higher privacy. As suggested by [5], we combine the privacy metrics into an overall average privacy score defined as:

$$\begin{aligned} \text{Pr. score} = \frac{1}{6}\Big(& (1 - |\text{priv. loss}_{\text{NNAA}}|) + (1 - |\text{priv. loss}_{\text{NNDR}}|) \\ & + (1 - \text{hit rate}) + (1 - \epsilon\text{-risk}) + (1 - \text{MIA}) + (1 - \text{ADR})\Big). \end{aligned} \tag{A.3}$$

As for the utility, the mutual information matrices of the real and synthetic data can both be used for visualization and also be converted to a single number by taking the Frobenius norm of the difference matrix[3]. Here, a smaller value indicates that the mutual information between the attributes is similar between the datasets. To evaluate if the marginal distributions of the attributes are significantly different between synthetic and real data, we used the Kolmogorov-Smirnov (KS) and Total Variation Distance (TVD) tests. The KS-test, used for numerical attributes, can be interpreted as the maximum separation between two empirical cumulative distribution functions (ECDF). For categorical variables, SynthEval employs the TVD-test instead, which is an average of the absolute difference in the probability distributions. SynthEval combines these tests into an average and calculates the fraction of significant tests. For both, low values indicate that the synthetic data captures the true distributions[3]. SynthEval also includes a metric that evaluates four different machine learning-based classifiers' capability to classify real unseen samples. The metric is the average difference in F1 score, after five-fold cross-validation between the classifiers trained on real data versus the synthetic. Both the F1 scores on the validation folds ($F1_t rain$) and on the hold-out test set ($F1_t est$) are used. The classifiers used are scikit-learn's implementations of AdaBoost, decision tree, random forest, and logistic regression[7], and their performance is both presented as an average score following 5-fold cross-validation, and on a hold-out test set. Similarly, as with the privacy metrics, we computed an average utility score. Since the mutual information matrix difference is unbounded from above, the hyperbolic tangent function is applied [5].

$$\text{Ut. score} = \frac{1}{5}\Big((1 - \tanh(\text{MI diff.})) + (1 - \text{KS/TVD}_{\text{stat}}) + (1 - \text{KS/TVD}_{\text{frac}}) + (1 - \text{Avg. Diff. F1}_{\text{train}}) + (1 - \text{Avg. Diff. F1}_{\text{test}})\Big). \tag{A.4}$$

Table A.3: Metrics used from SynthEval, with short descriptions. The arrows indicate whether a higher or lower value is preferred.

**Name**	**Description**	**Range**	↑↓
UTILITY			
MI diff	The Frobenius norm of the Mutual Information Matrix Difference	$[0, \infty)$	↓
NNAA	Nearest Neighbour Adervsarial Acc.	$[0, 1)$	
KS-test	Kolmogorov-Smirnov test (p-value)	$(0, 1)$	↓
	KS-test fraction of significant tests	$[0, 1]$	↓
TVD-test	Total Variantion Diff. test for categorical attrs.	$(0, 1)$	↓
	TVD-test fraction of significant tests	$[0, 1]$	↓
Avg. Diff. F1	Average difference in F1 score for four classifiers (5-fold cross-validation)	$[0, 1)$	↓
	Average difference in F1 score (on test data)	$[0, 1)$	↓
PRIVACY			
NNAA Privacy loss	Difference in NNAA between train/synthetic and test	$[0, 1)$	↓
NNDR Privacy loss	Privacy loss NN Distance Ratio	$[0, 1)$	↓
Hitting rate		$[0, 1]$	↓
ϵ-identif. risk		$[0, 1]$	↓
MIA	Membership Inference Attack (Recall)	$[0, 1]$	↓
ADR	Attribute Disclosure Risk (Avg. acc.)	$[0, 1]$	↓

Appendix B. Supplementary results

Table B.4: Results of the hyperparameter search for CTGAN. We kept the number of training epochs found by the grid search for the default model, for comparable results.

**Model**	Epochs	Batch size	Generator		Discriminator	
			L. rate	Dim.	L. rate	Dim.
CTGAN (default)	750	500	2e-4	(256, 256)	2e-4	(256, 256)
CTGAN (optim)	750	50	0.0001	(64, 64)	0.0001	(32, 32)

Table B.5: Results of the hyperparameter search for TableDiffusion, by the Optuna library. We used runs of 50 trials over 100 epochs.

**Model**	ϵ	Cohort	Diffusion steps	Batch size	Generator dim.	L. rate
TableDiffusion	1	ADNI	5	250	(64,64)	0.011
TableDiffusion	10	ADNI	5	250	(128,128)	0.003
TableDiffusion	25	ADNI	5	100	(64,64)	0.016
TableDiffusion	50	ADNI	5	100	(128,128)	0.001
TableDiffusion	100	ADNI	5	250	(64,64)	0.013
TableDiffusion	1	A4	3	250	(256,256)	0.003
TableDiffusion	10	A4	3	100	(64,64)	0.001
TableDiffusion	25	A4	3	100	(64,64)	0.001
TableDiffusion	50	A4	3	100	(64,64)	0.001
TableDiffusion	100	A4	3	100	(64,64)	0.0002
TableDiffusion	1	ADNI+	5	500	(16,16)	0.013
TableDiffusion	10	ADNI+	5	500	(16,16)	0.013
TableDiffusion	25	ADNI+	5	500	(16,16)	0.013
TableDiffusion	50	ADNI+	5	500	(16,16)	0.047
TableDiffusion	100	ADNI+	5	500	(16,16)	0.047

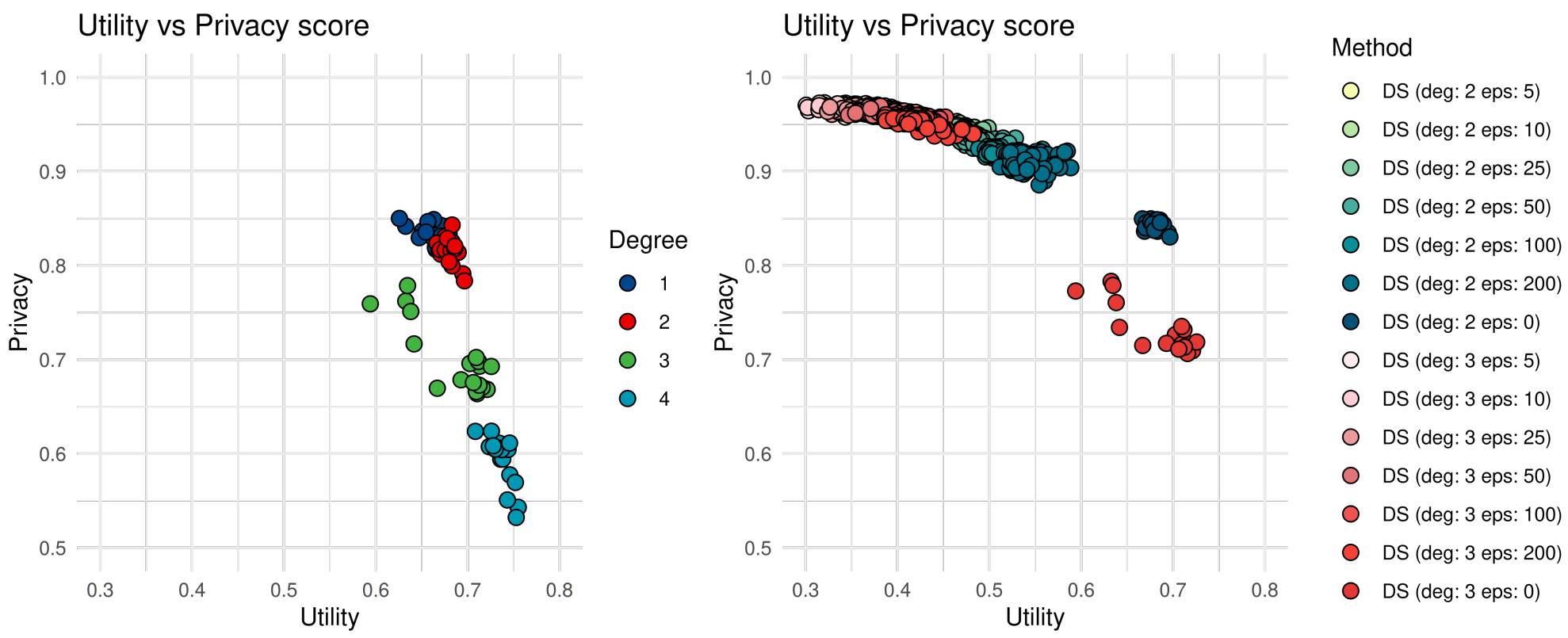

Figure B.1: Evaluation of the optimal degree of the Bayesian networks using the privacy and utility scores from SynthEval. Utility and privacy scores for the non-differentially private datasets ($\epsilon = 0$) with network degrees 1 to 4 (Left). Bayesian networks with degrees 2 and 3 constructed with differential privacy ($\epsilon = [200, 100, 50, 25, 10, 5]$) and without (Right).

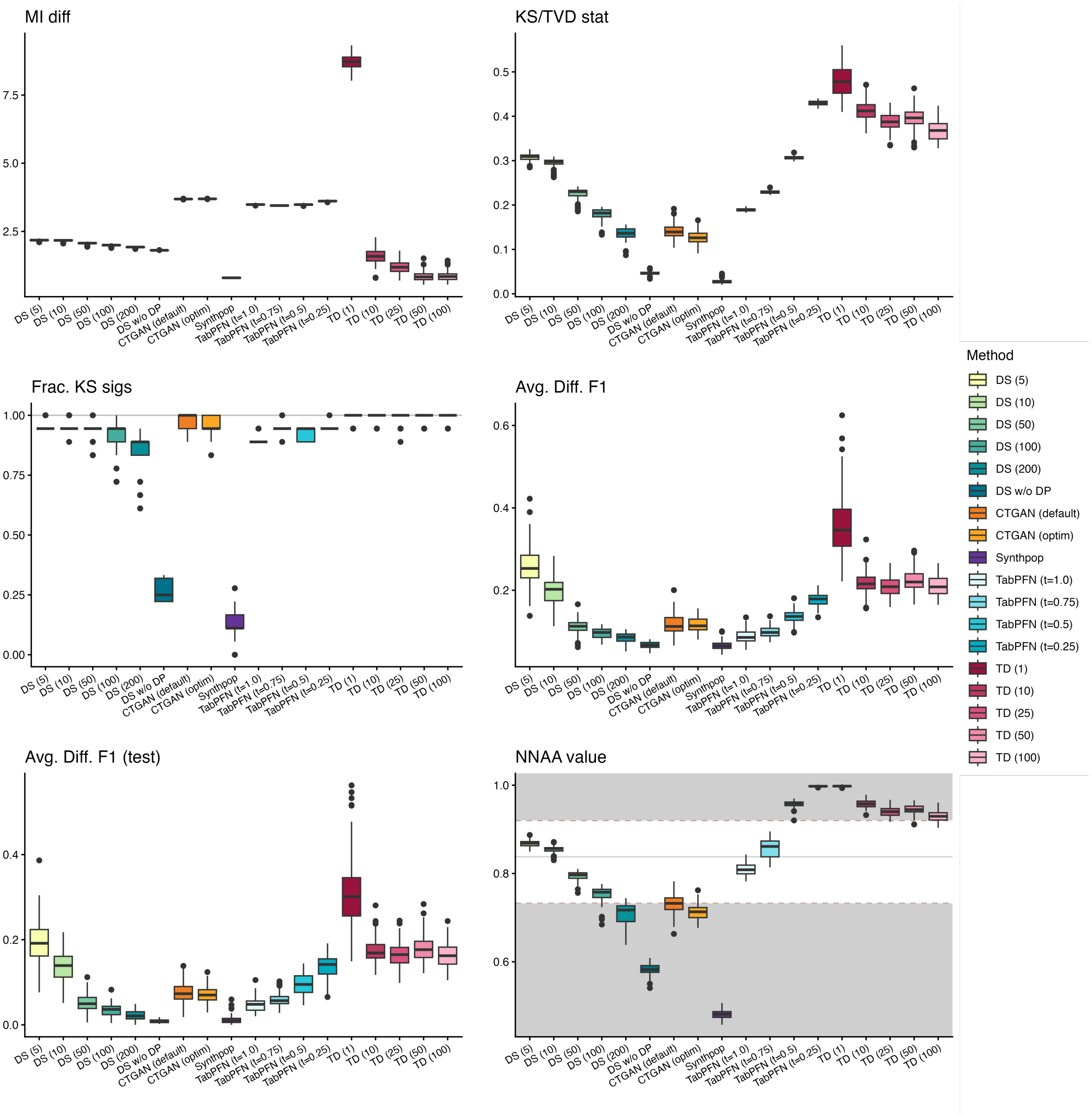

Figure B.2: Utility evaluation with SynthEval for the synthetic ADNI datasets generated using DataSynthesizer (DS) (with ϵ in parenthesis) and without differential privacy (DP), CTGAN (with number of epochs in parenthesis), TabPFN (sampling temperature in parenthesis) and Synthpop. For all metrics, a lower value corresponds with better utility. The boxplot visualizes median (middle line), the interquantile range (IQR) from the 25th to the 75th percentile (box), 1.5 · IQR (whiskers), and any outliers. For the DS datasets, summary statistics were estimated by the 100 constructed Bayesian networks for each epsilon setting (18 for the datasets without ϵ-differential privacy, ϵ = 0). For the CTGAN, TabPFN and Synthpop datasets, 100 different datasets generated with different seeds was used for statistical evaluation. The gray line and dashed red lines represent the global mean and 99% CI, estimated using a linear mixed-effects model with a random intercept for method to account for between-method variability.

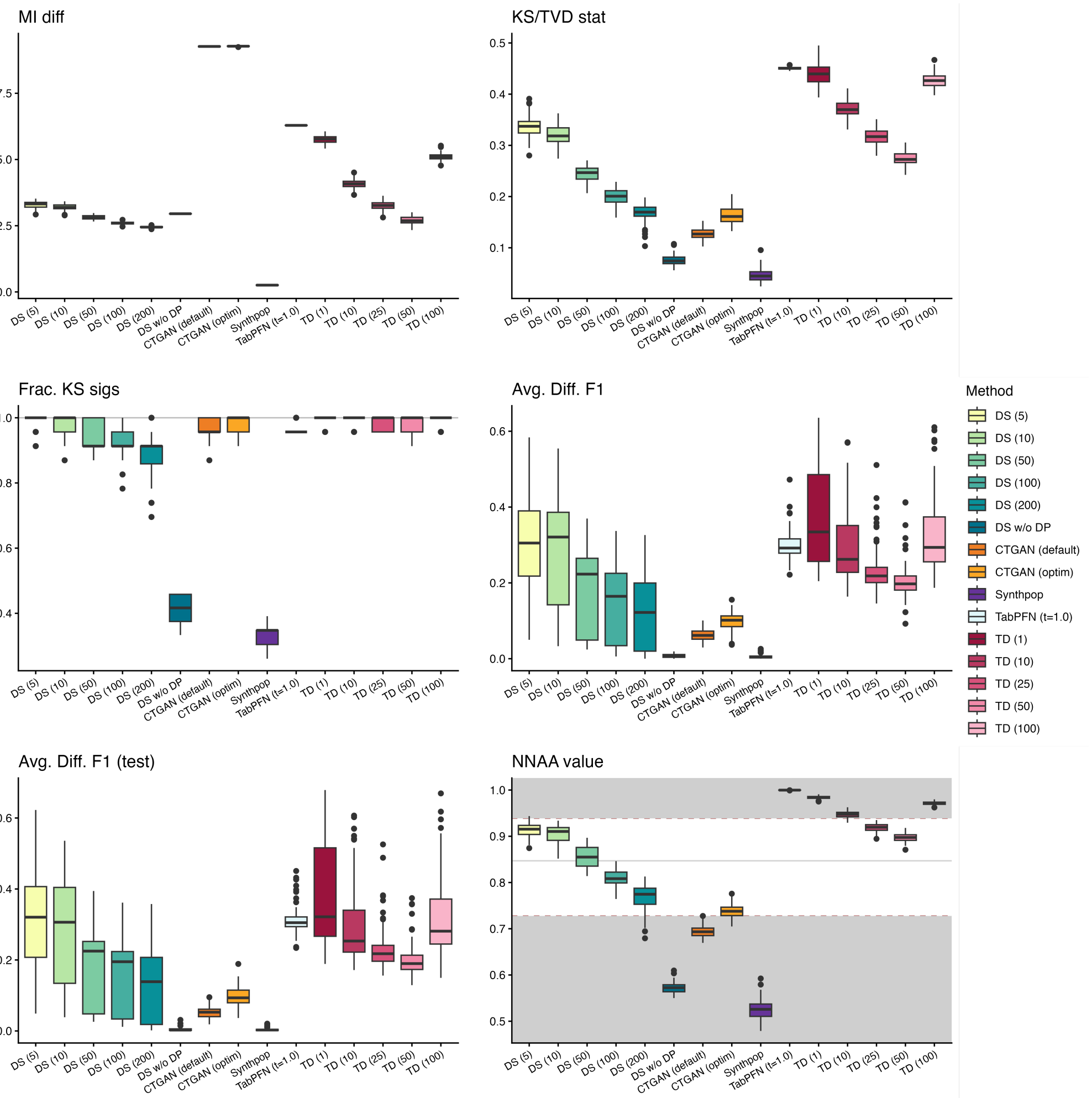

Figure B.3: Utility evaluation with SynthEval for the synthetic A4 datasets generated using DataSynthesizer (DS) (with ϵ in parenthesis) and without differential privacy (DP), CTGAN (with number of epochs in parenthesis), TabPFN (sampling temperature in parenthesis) and Synthpop. For all metrics, a lower value corresponds with better utility. The boxplot visualizes median (middle line), the interquantile range (IQR) from the 25th to the 75th percentile (box), $1.5 \cdot$ IQR (whiskers), and any outliers. For the DS datasets, summary statistics were estimated by the 100 constructed Bayesian networks for each epsilon setting (18 for the datasets without ϵ-differential privacy, $\epsilon = 0$). For the CTGAN and Synthpop datasets, 100 different datasets generated with different seeds was used for statistical evaluation. The gray line and dashed red lines represent the global mean and 99% CI, estimated using a linear mixed-effects model with a random intercept for method to account for between-method variability.

Table B.6: Resulting hyperparameters after 6-fold cross-validation grid search of the SVM models (top) and HGBoost (bottom) on the four different tasks. This was done on the real training dataset. C - Regularization parameter, γ - Kernel coefficient.

**Task (SVM)**	Cohort	Kernel	C	γ	ϵ-SVR param.
Diagnosis	ADNI	Radial Basis Function	10	$1/n_{\text{features}}$	-
Aβ	ADNI	Radial Basis Function	10	$1/n_{\text{features}}$	-
Aβ	A4	Radial Basis Function	10	$1/n_{\text{features}}$	-
ADAS13	ADNI	Radial Basis Function	100	$1/n_{\text{features}}$	0.01
MMSE	ADNI	Radial Basis Function	50	$1/n_{\text{features}}$	1
MMSE	A4	Radial Basis Function	2.5	$1/n_{\text{features}}$	1

**Task (HGBoost)**	Cohort	L2 reg.	Learning rate	Max bins	Max depth	Max iter.	Max leaf nodes	Min samples
Diagnosis	ADNI	1	0.01	255	3	300	31	100
Aβ	ADNI	0	0.2	125	3	100	31	100
Aβ	A4	0	0.01	255	3	100	31	20
ADAS13	ADNI	0	0.1	125	None	100	31	100
MMSE	ADNI	10	0.1	125	3	200	31	100
MMSE	A4	10	0.01	125	3	300	31	50

References

[1] H. Ping, J. Stoyanovich, B. Howe, DataSynthesizer: Privacy-preserving synthetic datasets, in: Proceedings of the 29th International Conference on Scientific and Statistical Database Management, SSDBM '17, Association for Computing Machinery, pp. 1–5. doi:10.1145/3085504.3091117.
URL `https://dl.acm.org/doi/10.1145/3085504.3091117`

[2] J. Zhang, G. Cormode, C. M. Procopiuc, D. Srivastava, X. Xiao, PrivBayes: Private data release via bayesian networks 42 (4) 1–41. doi:10.1145/3134428.
URL `https://dl.acm.org/doi/10.1145/3134428`

[3] A. D. Lautrup, T. Hyrup, A. Zimek, P. Schneider-Kamp, Syntheval: A framework for detailed utility and privacy evaluation of tabular synthetic data, arXiv (2024). doi:10.48550/arXiv.2404.15821.

[4] A. Yale, S. Dash, R. Dutta, I. Guyon, A. Pavao, K. P. Bennett, Generation and evaluation of privacy preserving synthetic health data, Neurocomputing 416 (2020) 244–255. doi:10.1016/j.neucom.2019.12.136.
URL `https://doi.org/10.1016/j.neucom.2019.12.136`

[5] A. Lautrup, T. Hyrup, A. Zimek, P. Schneider-Kamp, Systematic review of generative modelling tools and utility metrics for fully synthetic tabular data, ACM Comput. Surv. (2024). doi:doi.org/10.1145/3704437.

[6] J. Yoon, L. N. Drumright, M. van der Schaar, Anonymization through data synthesis using generative adversarial networks (ads-gan), IEEE Journal of Biomedical and Health Informatics 24 (8) (2020) 2378–2388. doi:10.1109/JBHI.2020.2980262.

[7] F. Pedregosa, G. Varoquaux, A. Gramfort, V. Michel, B. Thirion, O. Grisel, M. Blondel, P. Prettenhofer, R. Weiss, V. Dubourg, J. Vanderplas, A. Passos, D. Cournapeau, M. Brucher, M. Perrot, E. Duchesnay, Scikit-learn: Machine learning in Python, Journal of Machine Learning Research 12 (2011) 2825–2830.
